# New Lipidyl-Cyclodextrins Obtained by Ring Opening of Methyl Oleate Epoxide Using Ball Milling

**DOI:** 10.3390/biom10020339

**Published:** 2020-02-20

**Authors:** Estefania Oliva, David Mathiron, Sébastien Rigaud, Eric Monflier, Emmanuel Sevin, Hervé Bricout, Sébastien Tilloy, Fabien Gosselet, Laurence Fenart, Véronique Bonnet, Serge Pilard, Florence Djedaini-Pilard

**Affiliations:** 1 LG2A UMR CNRS 7378, Université de Picardie Jules Verne, 80039 Amiens CEDEX, France; estefania.oliva@etud.u-picardie.fr (E.O.); veronique.bonnet@u-picardie.fr (V.B.); 2 Plateforme Analytique, Université de Picardie Jules Verne, 80039 Amiens CEDEX, France; david.mathiron@u-picardie.fr (D.M.); sebastien.rigaud@etud.u-picardie.fr (S.R.); serge.pilard@u-picardie.fr (S.P.); 3 Univ. Artois, CNRS, Centrale Lille, Univ. Lille, UMR 8181–UCCS–Unité de Catalyse et Chimie du Solide, F-62300 Lens, France; eric.monflier@univ-artois.fr (E.M.); herve.bricout@univ-artois.fr (H.B.); sebastien.tilloy@univ-artois.fr (S.T.); 4 LBHE EA 2465, Université d’Artois, 62307 Lens CEDEX, France; emmanuel.sevin@univ-artois.fr (E.S.); fabien.gosselet@univ-artois.fr (F.G.); laurence.tilloy@univ-artois.fr (L.F.)

**Keywords:** host-guest chemistry, cyclodextrin, sustainability, ball milling, mass spectrometry

## Abstract

Bearing grafts based on fatty esters derivatives, lipidyl-cyclodextrins (L-CDs) are compounds able to form water-soluble nano-objects. In this context, bicatenary biobased lipidic-cyclodextrins of low DS were easily synthesized from a fatty ester epoxide by means of alternative methods (ball-milling conditions, use of enzymes). The ring opening reaction of methyl oleate epoxide needs ball-milling and is highly specific of cyclodextrins in solventless conditions. L-CDs are thus composed of complex mixtures that were deciphered by an extensive structural analysis using mainly mass spectrometry and NMR spectroscopy. In addition, as part of their potential use as vectors of active drugs, these products were submitted to an integrity study on in vitro model of the blood-brain-barrier (BBB) and the intestinal epithelium. No toxicity has been observed, suggesting that applications for the vectorization of active ingredients can be expected.

## 1. Introduction

Because of their cyclic structure, cyclodextrins (CDs) are able to accommodate hydrophobic molecules in their cavity, resulting in inclusion complexes. This property is at the origin of their many applications in various fields such as the pharmaceutical industry, cosmetics, hygiene, food processing, and chemistry, through catalysis and chromatographic techniques [1,2]. In addition, chemical modifications of their hydroxyl groups cause changes in their physico-chemical properties (solubility, self-assembly capacity or inclusion properties), thus increasing their fields of application. Moreover, it is well documented that amphiphilic CDs, carrying lipophilic groups, may have self-organizing properties as well as complexing properties with the invited molecules, improving their vectorization [3]. For several years, we have synthesized monosubstituted cyclodextrins-bearing hydrophobic anchors such as cholesterol [4], phospholipidyl [5], or glycerolipidyl [6] that are inserted into lipid membranes [7]. These properties are directly related to the type of cyclodextrin and lipidic moieties and also to the substitution degree (DS). Nevertheless, despite their potential interest in the vectorization of active ingredients, the synthesis of these amphiphilic CDs is both tricky and expensive, limiting any application. To illustrate, phospholipidyl and glycerolipidyl cyclodextrin derivatives [5,6] have been obtained in no less than seven steps of synthesis and two steps of purification with overall yields between 5% and 10%.

In this context, we have developed an easy pathway for lipidyl-cyclodextrins (L-CDs) synthesis with controlled DS. The self-assembly capacities of these L-CDs have been also evaluated in order to consider applications in the vectorization of active ingredients. The synthesized molecules are bicatenary L-CDs of low DS. The lipid part is derived from methyl oleate, a fatty acid methyl ester (FAME) easily accessible from triolein triglyceride, a major component of very high oleic sunflower oil (VHOSO). The grafting has been done by ring opening reaction of the methyl oleate epoxide by the CDs. The different strategies envisaged to carry out the opening reaction of the epoxide obtained by CDs is described. Indeed, activation modes such as ultrasound [8,9], microwaves [10,11], or mechanochemistry [12] are considered as useful green tools for the chemical modification of cyclodextrins. Cravotto et al. reported some works where ball milling is used to obtain well-known CD derivatives but under green and solventless conditions and with better yields [13,14]. Very few examples of more specific regioselectivity induced by mechanochemistry are reported. Menuel et al. described a very efficient method for obtaining mono-2-tosyl-β-CD and mono-(2,3-manno-epoxide)-β-CD [15]. Formation of inclusion complex in solid state between CD and TSIm is suggested to explain CD substitution at the secondary hydroxyl group. Here we focus on the results obtained (yields, DS) with the selected and optimized method using ball-milling conditions on the opening reaction of methyl oleate epoxide by cyclodextrins.

Single-step synthesis routes, overcoming protection and deprotection steps and allowing quantities of products to be reached at gram scale, are favored in order to facilitate the potential applications. However, the absence of protection and deprotection steps should lead to complex mixtures of diversely substituted CDs. Since the composition of these mixtures has an influence on the self-organizing capacity of the products, it is important to characterize it. This is why the implementation of an accurate characterization is fundamental and a real challenge in our work. It is well-known that complex CDs derivatives mixtures lead to broad and unresolved ^1^H-NMR signals [16] preventing the attribution of their inextricable spectra. In this context, NMR is of only limited help and mass spectrometry is considered as the most appropriate technique. Indeed, electrospray ionization (ESI) either in direct introduction [17] or coupled with liquid chromatography (LC/ESI-MS) [18] and matrix assisted laser desorption ionization (MALDI) [16] are frequently used in that context. To go deeper in the structural analysis of cyclic oligosaccharides tandem mass spectrometry (MS/MS) [19] and more recently ion mobility spectrometry (IMS) [20] are described. In this study, in addition to classical NMR and mass spectrometry experiments, we focus on an original approach based on ion mobility measurements for a better structural characterization of L-CDs isomers.

Finally, we used two recognized in vitro models of human intestinal and blood-brain barriers to evaluate the toxicity of different concentrations of L-CDs. No toxicological or deleterious effects have been observed.

## 2. Materials and Methods

### 2.1. Chemicals

Native β-CD and RAMEB were obtained from Wacker Chemicals. Native α-CD and γ-CD, HP-α-CD, HP-β-CD, HP-γ-CD, methyl oleate (Methyl-*cis*-9-octadecenoate, 99% purity) and immobilized lipase (lipase acrylic resin from *Candida antarctica*, ≥5000 U/g) were supplied by Sigma (St. Louis, MO, USA). Other chemicals were purchased from Sigma and Acros. Deuterated solvents were purchased from Eurisotop.

### 2.2. Flash Chromatography

A flash chromatography apparatus (Reveleris iES Flash System) provided by Grace has been used for the purification of CD(C_9_)_2_OOMe. The separation was carried out using a Grace Reveleris C18 40 μm, 40 g column. A gradient of H_2_O/MeOH (from 80:20 to 0:100 (*v*/*v*)) in 15 min was applied to allow removal of the remaining native β-CD and obtaining the desired product. The detection of the compound is carried out with an evaporative light scattering detector.

### 2.3. Ball Milling 

Syntheses of CD(C_9_)_2_OOMe were carried out in a SPEX Mixer Mill 8000M vibration ball mill. For this, we used a 65 mL screw-capped stainless steel jar containing 12 stainless steel balls of diameter between 4.0 and 12.7 mm (3 balls of 12.7 mm, 5 of 6.4 mm, and 4 of 4.0 mm). The vessel was shaken at a frequency of about 18 Hz, describing a “∞” for the duration of the reaction (5 cycles of 1 h).

### 2.4. Mass Spectrometry Analysis

Electrospray high-resolution mass spectrometry experiments (ESI-HRMS) was performed on a SYNAPT G2-Si-Q-TOF hybrid quadrupole time-of-flight instrument (Waters, Manchester, UK), hyphenated with an UHPLC system (ACQUITY UPLC H-Class) and a traveling wave ion mobility (TWIM) cell. The ESI source was operated in the positive ionization mode using a capillary voltage of +3 kV and the following conditions: cone voltage, 120 V; source offset, 20 V; source temperature, 120 °C; desolvation gas temperature, 450 °C; desolvation gas flow, 800 L/h; and cone gas flow, 50 L/h. Nitrogen (>99.5%) was employed as the desolvation gas. For negative ionization mode a capillary voltage of −2.5 kV was used. Mass calibration was carried out using a sodium formate solution (10 mM NaOH in isopropanol/water/formic acid 49.9:49.9:0.2, *v*/*v*/*v*) and the lock mass correction was applied for accurate mass measurements using protonated and deprotonated molecules ([M + H]^+^ at *m*/*z* 556.2771 in ESI^+^ and [M − H]^−^ at *m*/*z* 554.2615 in ESI^−^) formed from a Leu-enkephalin solution (1 ng/µL in H_2_O/CH_3_CN/formic acid 50:49.9:0.1, *v*/*v*/*v*). The scan range was *m*/*z* 50–2500 at 0.25 s/scan. The TOF was operated in the resolution mode, providing an average resolving power of 25,000 (FWHM). For MS/MS, spectra were recorded using the trap cell, with a collision energy set to 95 eV under Argon (99.9999%) as the collision gas. All spectra were recorded in the continuum mode. Data acquisition was performed with MassLynx software (V4.1, Waters).

Direct introduction and UHPLC conditions: Samples were prepared in MeOH at a concentration of 1 mg/mL and 1 µL was injected. Automated flow injections (FIA) were performed using MeOH as the mobile phase with a flow rate of 0.4 mL/min. For UHPLC separations an ACQUITY UPLC BEH C8 (100 × 2.1 mm, 1.8 µm) column from Waters, maintained at 40 °C, was used. The elution was performed using a 0.4 mL/min mobile phase gradient of water (A) and methanol (B), programmed as follow (A:B): 60:40 (t = 0 min), 20:80 (t = 10 min), 20:80 (t = 11 min), 0:100 (t = 12 min), 0:100 (t = 15 min), 60:40 (t = 16 min), 60:40 (t = 20 min).

Ion mobility: Ion mobility separation has been carried out by activating TWIM cell operating under nitrogen (>99.5%) with wave height (WH), wave velocity (WV), and gas flow values at 40 V, 550 m/s, and 90 mL/min, respectively. The mass of β-CD(C_9_)_2_OOMe DS = 1 ([M + Na]^+^ *m/z* 1469.6254) was previously selected by the quadrupole to reduce noise and for more specificity. External collision cross section (CCS) calibration was performed using a polyalanine solution (50 µM in water/acetonitrile/formic acid 49.95:49.95:0.1, *v*/*v*/*v*). The arrival time (AT) of each DS = 1 isomer was extracted with Driftscope software (V2.8, Waters). Molecules were simulated by Chem3D (V15.1, PerkinElmer) and CCS values were predicted through IMoS software (V1.09c, https://www.imospedia.com).

### 2.5. NMR Analysis

The NMR experiments were carried out on 2 Bruker AVANCE spectrometers (400 and 600 MHz). They were therefore performed respectively at 400 MHz for ^1^H and 101 MHz for ^13^C, or 600 MHz for ^1^H and 151 MHz for ^13^C. The deuterated solvents used were pyridine, chloroform, water, or methanol. The chemical shifts (δ) are expressed in ppm relative to the residual signal of the non-deuterated solvent as internal reference. The spectra were recorded at 298 K. The length of the 90° pulse was approximately 7 μs. 1D NMR data spectra were collected using 16K data points. 2D experiments were run using 1K data points and 512 time increments. The phase sensitive (TTPI) sequence was used and processing resulted in a 1 K × 1 K (real-real) matrix. Details of the experimental conditions are given in the figure captions. For diffusion coefficient measurements, 2D ^1^H DOSY NMR experiments were carried out using the Bruker sequence ledbpgp2s.

### 2.6. Synthesis and Product Characterization

#### 2.6.1. Methyl Oleate Epoxide

Preparation of epoxidized methyl oleate was carried out by reacting methyl oleate with 0.4 eq. formic acid and hydrogen peroxide (35%w aq., 6 eq.) from 0 to 40 °C for 16 h. After basic work-up, epoxide was obtained with 98% yield (yellow oil) [21]. ¹H NMR (400 MHz, CDCl_3_) δ (ppm): 3.65 (s, 3H, OCH_3_), 2.96–2.82 (m, 2H, H_9_ and H_10_), 2.31–2.27 (t, 2H, *J* = 7.5 Hz, CH_2_COOCH3), 1.71–1.55 (m, 2H, CH_2_), 1.55–0.96 (m, 24H, CH_2_), 0.87 (t, 3H, *J* = 6.9 Hz, CH_3_). ^13^C NMR (101 MHz, CDCl_3_) δ (ppm): 174.4 (C=O), 57.4, 57.3 (C-9 and C-10), 51.6 (C-OCH_3_), 34.2 (CH_2_ (CH_2_OCO)), 32.0–22.8 (CH_2_ (alkyl chains)), 14.2 (CH_3_). MS (ESI): *m*/*z* 335.2578 ([M + Na]^+^).

#### 2.6.2. CD(C_9_)_2_OOMe

General procedure: All the CDs were lyophilized before use. Methyl oleate epoxide (275 mg, 0.88 mmol, 0.5 eq) and H_2_SO_4_ (4.7 μL, 0.088 mmol, 0.05 eq) were vortexed and added to the lyophilized CD (1 eq, 1.76 mmol). The reaction was carried out for 1 h in a ball mill. Then, a mixture of the methyl oleate epoxide (275 mg, 0.88 mmol, 0.5 eq) and H_2_SO_4_ (4.7 μL, 0.088 mmol, 0.05 eq) was added to the reaction mixture. The reaction continued for 1 h again. This addition was repeated three times; so that 1 eq of CD reacted with a total of 2.5 eq of epoxide and 0.25 eq of H_2_SO_4_ for a total duration of 5 h. The solid was recovered with 20 mL of DMF. The product was then concentrated in a rotary evaporator and poured dropwise into the cyclohexane with stirring to precipitate it. Centrifugation was carried out and the final product was obtained after purification by reverse phase flash chromatography (40 μm C18 column, 40 g) with a H_2_O/MeOH gradient (from 80:20 to 0:100 (*v*/*v*)) for 15 min.

- β*-CD(C_9_)_2_OOMe, **1:*** Yield: 55%**.** ¹H NMR (600 MHz, Pyridine-*d*_5_) δ (ppm): 5.69–5.60 (H_1_), 4.85–3.55 (H_2_, H_3_, H_4_, H_5_, H_6_), 3.63 (-OCH_3_), 2.30–1.01 (CH_2_ (alkyl chains)), 0.84 (CH_3_). ^13^C NMR (151 MHz, Pyridine-*d*_5_) δ (ppm): 174.4 (C=O), 104.3 (C_1_), 85.2–83.0 (C_4_), 75.3–72.7 (C_2_, C_3_, C_5_), 62.4–61.6 (C_6_), 51.7 (-OCH_3_), 34.5–23.0 (CH_2_ (alkyl chains)), 14.6 (CH_3_). HRMS (ESI): *m*/*z* DS1: 1469.6233 ([M + Na]^+^ (C_61_H_106_O_38_Na requires 1469.6260)), *m*/*z* DS2: 1781.8888 ([M + Na]^+^ (C_80_H_142_O_41_Na requires 1781.8924)) SI Figure S1.
- α*-CD(C_9_)_2_OOMe, **2:*** Yield: 28%**.** ¹H NMR (600 MHz, Pyridine-*d*_5_) δ (ppm): 5.77–5.38 (H_1_), 5.01–3.49 (H_2_, H_3_, H_4_, H_5_, H_6_), 2.66–1.06 (CH_2_ (alkyl chains)), 0.84 (CH_3_). ^13^C NMR (151 MHz, Pyridine-*d*_5_) δ (ppm): 174.3 (C=O), 104.5–103.5 (C_1_), 86.1–82.4 (C_4_), 76.4–70.0 (C_2_, C_3_, C_5_), 62.4–60.8 (C_6_), 51.6 (-OCH_3_), 35.1–22.7 (CH_2_ (alkyl chains)), 14.6 (CH_3_). HRMS (ESI): *m*/*z* DS1: 1307.5717 ([M + Na]^+^ (C_55_H_96_O_33_Na requires 1307.5732)), *m*/*z* DS2: 1619.8401 ([M + Na]^+^ (C_74_H_132_O_36_Na requires 1619.8396)) SI Figure S2.
- γ*-CD(C_9_)_2_OOMe, **3:*** Yield: 33%**.** ¹H NMR (600 MHz, Pyridine-*d*_5_) δ (ppm): 5.98–5.45 (H_1_), 4.79–3.52 (H_2_, H_3_, H_4_, H_5_, H_6_), 3.63 (-OCH_3_), 2.50–1.06 (CH_2_ (alkyl chains)), 0.81 (CH_3_). ^13^C NMR (151 MHz, Pyridine-*d*_5_) δ (ppm): 174.2 (C=O), 103.6 (C_1_), 85.5–81.0 (C_4_), 75.6–71.3 (C_2_, C_3_, C_5_), 63.2–60.9 (C_6_), 51.6 (-OCH_3_), 35.0–22.6 (CH_2_ (alkyl chains)), 14.6 (CH_3_). HRMS (ESI): *m*/*z* DS1: 1631.6769 ([M + Na]^+^ (C_67_H_116_O_43_Na requires 1631.6788)), *m*/*z* DS2: 1943.9395 ([M + Na]^+^ (C_86_H_152_O_46_Na requires 1943.9452)) SI Figure S3.
- HP-α*-CD(C_9_)_2_OOMe, **4:*** Yield: 33%**.** ¹H NMR (600 MHz, Pyridine-*d*_5_) δ (ppm): 6.42–5.24 (H_1_), 4.92–3.55 (H_2_, H_3_, H_4_, H_5_, H_6_), 3.63 (-OCH_3_), 2.43–1.46 (CH_2_ (alkyl chains)), 1.50–1.03 (CH_3_ (hydroxypropyl groups)), 0.84 (CH_3_ (alkyl chains)). ^13^C NMR (151 MHz, Pyridine-*d*_5_) δ (ppm): 174.3 (C=O), 105.0–100.5 (C_1_), 86.1–77.4 (C_4_), 76.0–66.0 (C_2_, C_3_, C_5_), 63.0–60.7 (C_6_), 51.6 (-OCH_3_), 34.8–22.9 (CH_2_ (alkyl chains)), 21.0–19.9 (CH_3_ (hydroxypropyl groups)), 14.6 (CH_3_). HRMS (ESI): *m*/*z* DS1: 1597.7808 ([M + Na]^+^ (C_70_H_126_O_38_Na requires 1597.7825)), *m*/*z* DS2: 1852.0050 ([M + Na]^+^ (C_86_H_156_O_40_Na requires 1852.0071)) SI Figure S4.
- HP-β*-CD(C_9_)_2_OOMe, **5:*** Yield: 35%. ¹H NMR (600 MHz, Pyridine-*d*_5_) δ (ppm): 6.22–5.35 (H_1_), 4.80–3.55 (H_2_, H_3_, H_4_, H_5_, H_6_), 3.62 (-OCH_3_), 2.50–1.46 (CH_2_ (alkyl chains)), 1.50–1.10 (CH_3_ (hydroxypropyl groups)), 0.83 (CH_3_ (alkyl chains)). ^13^C NMR (151 MHz, Pyridine-*d*_5_) δ (ppm): 174.3 (C=O), 105.0–100.0 (C_1_), 85.4–78.0 (C_4_), 75.7–66.2 (C_2_, C_3_, C_5_), 62.4–61.3 (C_6_), 51.6 (-OCH_3_), 35.0–23.1 (CH_2_ (alkyl chains)), 21.1–19.9 (CH_3_ (hydroxypropyl groups)), 14.6 (CH_3_). HRMS (ESI): *m*/*z* DS1: 1875.9177 ([M + Na]^+^ (C_82_H_148_O_45_Na requires 1875.9190)), *m*/*z* DS2: 2188.1821 ([M + Na]^+^ (C_101_H_184_O_48_Na requires 2188.1855)), *m*/*z* DS3: 2500.4465 ([M + Na]^+^ (C_120_H_220_O_51_Na requires 2500.4519)). SI Figure S5.
- HP-γ*-CD(C_9_)_2_OOMe, **6:*** Yield: 44%**.** ¹H NMR (600 MHz, Pyridine-*d*_5_) δ (ppm): 6.49–5.31 (H_1_), 4.70–3.49 (H_2_, H_3_, H_4_, H_5_, H_6_), 3.63 (-OCH_3_), 2.50–1.46 (CH_2_ (alkyl chains)), 1.44–1.01 (CH_3_ (hydroxypropyl groups)), 0.82 (CH_3_ (alkyl chains)). ^13^C NMR (151 MHz, Pyridine-*d*_5_) δ (ppm): 174.3 (C=O), 104.6–98.9 (C_1_), 86.0–78.4 (C_4_), 75.8–65.6 (C_2_, C_3_, C_5_), 63.0–60.2 (C_6_), 51.7 (-OCH_3_), 34.9–22.7 (CH_2_ (alkyl chains)), 20.9–19.6 (CH_3_ (hydroxypropyl groups)), 14.6 (CH_3_). HRMS (ESI): *m*/*z* DS1: 1979.9336 ([M + Na]^+^ (C_85_H_152_O_49_Na requires 1979.9300)), *m*/*z* DS2: 2292.1956 ([M + Na]^+^ (C_104_H_188_O_52_Na requires 2292.1964)), *m*/*z* DS3: 2604.4609 ([M + Na]^+^ (C_123_H_224_O_55_Na requires 2604.4629)) SI Figure S6.

#### 2.6.3. CD(C_9_)_2_OOH

General procedure: Immobilized lipase from *Candida Antarctica* was added to a suspension of CD(C_9_)_2_OOMe (0.4 mmol, 1 eq) in water (90 mL). The reaction took place in a rotary evaporator at 90 mbar and 23 °C. The duration of the reaction depends on the starting material. The product was isolated by simple filtration of the immobilized lipase followed by the lyophilization to obtain the derivative CD(C_9_)_2_OOH.

- α*-CD(C_9_)_2_OOH, **7**:* Yield: 78%. ¹H NMR (600 MHz, Pyridine-d_5_) δ (ppm): 5.77–5.38 (H_1_), 5.01–3.49 (H_2_, H_3_, H_4_, H_5_, H_6_), 2.66–1.06 (CH_2_ (alkyl chains)), 0.84 (CH_3_). ^13^C NMR (151 MHz, Pyridine-*d*_5_) δ (ppm): 176.7 (C=O), 104.7–103.1 (C_1_), 85.7–81.9 (C_4_), 76.3–69.4 (C_2_, C_3_, C_5_), 62.7–60.8 (C_6_), 35.9–22.9 (CH_2_ (alkyl chains)), 14.6 (CH_3_). HRMS (ESI): *m*/*z*: 1293.5549 ([M + Na]^+^ (C_54_H_94_O_33_Na requires 1293.5575)) SI Figure S7.
- β*-CD(C_9_)_2_OOH, **8:*** Yield: 69%. ¹H NMR (600 MHz, Pyridine-d_5_) δ (ppm): 5.67(H_1_), 4.97–3.49 (H_2_, H_3_, H_4_, H_5_, H_6_), 2.56–1.01 (CH_2_ (alkyl chains)), 0.81 (CH_3_).^13^C NMR (151 MHz, Pyridine-d_5_) δ (ppm): 176.5 (C=O), 104.3 (C_1_), 85.4–82.7 (C_4_), 75.5–72.2 (C_2_, C_3_, C_5_), 62.5–61.2 (C_6_), 35.4–22.9 (CH_2_ (alkyl chains)), 14.6 (CH_3_). HRMS (ESI): *m*/*z*: 1455.6115 ([M + Na]^+^ (C_60_H_104_O_38_Na requires 1455.6103)) SI Figure S8.
- γ*-CD(C_9_)_2_OOH, **9:*** Yield: 89%**.** ¹H NMR (600 MHz, Pyridine-*d*_5_) δ (ppm): 6.03–5.20 (H_1_), 5.10–3.47 (H_2_, H_3_, H_4_, H_5_, H_6_), 2.67–1.00 (CH_2_ (alkyl chains)), 0.84 (CH_3_). ^13^C NMR (151 MHz, Pyridine-*d*_5_) δ (ppm): 176.6 (C=O), 105.2–94.6 (C_1_), 85.2–78.2 (C_4_), 76.7–68.6 (C_2_, C_3_, C_5_), 64.9–60.2 (C_6_), 36.0–22.4 (CH_2_ (alkyl chains)), 14.6 (CH_3_). HRMS (ESI): *m*/*z*: 1617.6648 ([M + Na]^+^ (C_66_H_114_O_43_Na requires 1617.6632)) SI Figure S9.
- HP-α*-CD(C_9_)_2_OOH, **10:*** Yield: 82%**.** ¹H NMR (600 MHz, Pyridine-*d*_5_) δ (ppm): 5.72–5.31 (H_1_), 4.89–3.31 (H_2_, H_3_, H_4_, H_5_, H_6_), 2.78–1.47 (CH_2_ (alkyl chains)), 1.50–1.00 (CH_3_ (hydroxypropyl groups)), 0.84 (CH_3_ (alkyl chains)). ^13^C NMR (151 MHz, Pyridine-*d*_5_) δ (ppm): 176.7 (C=O), 105.2–100.0 (C_1_), 87.3–76.8 (C_4_), 76.6–65.1 (C_2_, C_3_, C_5_), 63.8–60.4 (C_6_), 36.6–22.4 (CH_2_ (alkyl chains)), 21.5–18.8 (CH_3_ (hydroxypropyl groups)), 14.6 (CH_3_). HRMS (ESI): *m*/*z*: 1525.7257 ([M + Na]^+^ (C_66_H_118_O_37_Na requires 1525.7250)) SI Figure S10.
- HP-β*-CD(C_9_)_2_OOH, **11:*** Yield: 84%**.** ¹H NMR (600 MHz, Pyridine-*d*_5_) δ (ppm): 6.00–5.35 (H_1_), 5.09–3.09 (H_2_, H_3_, H_4_, H_5_, H_6_), 2.96–1.57 (CH_2_ (alkyl chains)), 1.56–1.00 (CH_3_ (hydroxypropyl groups)), 0.84 (CH_3_ (alkyl chains)). ^13^C NMR (151 MHz, Pyridine-*d*_5_) δ (ppm): 176.6 (C=O), 105.2–100.0 (C_1_), 85.6–77.5 (C_4_), 75.7–65.7 (C_2_, C_3_, C_5_), 63.1–60.2 (C_6_), 36.2–22.6 (CH_2_ (alkyl chains)), 21.3–19.8 (CH_3_ (hydroxypropyl groups)), 14.6 (CH_3_). HRMS (ESI): *m*/*z*: 1861.9044 ([M + Na]^+^ (C_81_H_146_O_45_Na requires 1861.9034)) SI Figure S11.
- HP-γ*-CD(C_9_)_2_OOH, **12:*** Yield: 89%. ¹H NMR (600 MHz, Pyridine-*d*_5_) δ (ppm): 5.99–5.27 (H_1_), 4.80–3.24 (H_2_, H_3_, H_4_, H_5_, H_6_), 2.60–1.47 (CH_2_ (alkyl chains)), 1.47–1.02 (CH_3_ (hydroxypropyl groups)), 0.83 (CH_3_ (alkyl chains)). ^13^C NMR (151 MHz, Pyridine-*d*_5_) δ (ppm): 176.5 (C=O), 104.4–98.8 (C_1_), 85.9–77.6 (C_4_), 76.2–65.7 (C_2_, C_3_, C_5_), 62.9–60.4 (C_6_), 35.7–22.8 (CH_2_ (alkyl chains)), 20.9–19.6 (CH_3_ (hydroxypropyl groups)), 14.6 (CH_3_). HRMS (ESI): *m*/*z*: 1965.9083 ([M + Na]^+^ (C_84_H_150_O_49_Na requires 1965.9143)) SI Figure S12.

### 2.7. In Vitro Models of Intestinal and Blood-Brain Barriers

For the human in vitro Blood-Brain Barrier (BBB) model, it required the collection of human umbilical cord blood. Infants’ parents signed an informed consent form, in compliance with the French legislation. The protocol was approved by the French Ministry of Higher Education and Research (CODECOH Number DC2011-1321). All experiments were carried out in accordance with the approved protocol. The human in vitro BBB model isolated and purified the hematopoietic stem cells from umbilical cord blood [22]. These cells are then differentiated into endothelial cells as previously described. After the differentiation step, cells are seeded on matrigel-coated transwell inserts (0.4 µm) in the presence of brain pericytes at the bottom of the wells [23]. Endothelial cell medium (ECM, Sciencell) was changed every other day. After 6 days of co-culture, endothelial cells display the major features of the BBB reported in vivo and are then named brain-like endothelial cells (BLECs).

The intestinal barrier model cultivated Caco-2 cells on a filter as we previously published [24]. Briefly, 0.4 × 10^5^ Caco-2 cells, at passage 026, were seeded on 25 cm² plastic flask and changed every second day with complete medium containing Dulbecco’s modified Eagle medium (DMEM) high glucose (4500 mg/L) with L-glutamine (584 mg/L) supplemented by 10% of fetal calf/bovine serum 1% of non-essential amino acids without L-glutamine and 1% of penicillin and streptomycin solution. Cells were then trypsinized after 3 days of incubation while they cover 80–90% of the flask and seeded at a density of 5 × 10^5^ in 75 cm² flasks in complete medium. After 5 to 6 days, Caco2 cells reach high cells density and are then passaged into transwell inserts (0.4 µm). Cells were seeded at 225,000 cells/cm² and cultivated for 21–25 days in complete medium. Media was renewed every second day.

### 2.8. Permeability Assessment

Permeability of small integrity marker, ^14^C-sucrose, across the monolayer of cells was measured in presence of 5, 10, 20, 25, 30, 40, and 50 µM of cyclodextrin or 10% DMSO used as positive control of biological barrier disruptions. Filters containing cells were placed in wells containing Ringer-Hepes solution (RH) (150 mM NaCl, 5.2 mM KCl, 2.2 mM CaCl_2_, 0.2 mM MgCl_2_.6H_2_O, 6 mM NaHCO_3_, 5 mM HEPES, pH: 7.4). After filters transfer, RH containing 1500 Bq/mL of ^14^C-sucrose was added to the upper compartment. An aliquot of 500 μL from the lower compartment at each time point and an aliquot of 20 μL from the initial solution of ^14^C-Sucrose was used for quantification (Tricarb liquid scintillation counter, Perkinelmer). After one-hour incubation, the permeability of ^14^C-sucrose was measured and expressed in Papp (apparent permeability for the intestinal barrier model) and Pe (permeability for the BBB model). The apparent permeability coefficient (*Papp* in cm/s) was determined according to the equation: *Papp* = *J*/*A* × *C*_0_. Where, *J* is the rate of appearance of compound in the apical (acceptor) compartment (in mol/s), *C*_0_ is the initial concentration of compound in the basolateral (donor) compartment (in mol/cm^3^), *A* is the surface area of the filter (in cm^2^). For the BLEC model, endothelial permeability coefficient (*Pe*) was calculated as described previously [25]. In this calculation method, both filter without cell permeability (PSf = insert filter + matrigel coating) and filter plus cell permeability (PSt = filter + collagen + BLECs were taken into account, according to the formula: 1/PSe = 1/PSt + 1/PSf where PS is the permeability × surface area product (in microliters per minute) obtained by dividing the volume cleared from the donor to the receiver compartment (in µL) by the duration of the experiment (i.e., 60 min).

## 3. Results and Discussion

### 3.1. Synthesis

**Scheme 1:**
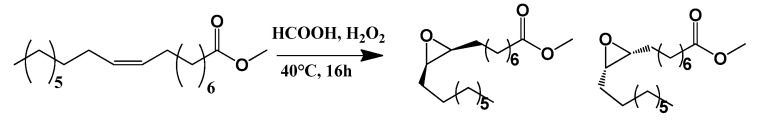
Epoxidation reaction of methyl oleate.

The main goal of this work is to synthesize new bicatenary cyclodextrins from substitution of native and modified cyclodextrins by fatty acid derivatives. In this way, we focused on the ring opening of the methyl oleate epoxide by the hydroxyl group of different cyclodextrins. The epoxidation reaction of pure methyl oleate (Methyl-cis-9-octadecenoate) was carried out according to the previously described conventional method using hydrogen peroxide and formic acid [21]. This resulted in methyl oleate epoxide with a yield of 98% (Scheme 1).

Epoxidation has been demonstrated by a ^1^H NMR analysis. Indeed, characteristic signals of the protons of the alkene group (5.38–5.28 ppm) disappeared, and the signals between 2.96 and 2.82 ppm, corresponding to the protons of the epoxide group were observed (SI, Figure S13). By electro spray ionization mass spectrometry in the positive ion mode (ESI^+^-MS), the formation of epoxide was also confirmed. Indeed, the [M + Na]^+^ ion of the starting olefin at *m*/*z* 319.26 was replaced by the one of the methyl oleate epoxide at *m*/*z* 335.2578 (+16 Da). Notice that the use of cis olefin leads to only cis epoxide derivatives, which is in agreement with the literature data [21].

In order to study the opening reaction of methyl oleate epoxide by CDs, we highlight some aspects to consider. On the one hand, very few examples in the literature have described the opening of epoxides by cyclodextrins. We can mention the etherification of β-CD with propylene oxide in the presence of NaOH to obtain the HP-β-CD derivative [26]. Reactions of β-CD with ethylene oxide, epichlorohydrin, or 1,4-butanediol diglycidyl ether in basic medium leading to water-soluble polymers have also been reported [27,28,29], and β-amino-alcohol derivatives of β-CDs were synthesized by reaction of amino β-CD and various epoxides [30]. It should be noted that most of the examples in the literature concerned reactions with highly reactive and low molecular weight epoxides and did not lead to the production of amphiphilic CDs. To date, the opening reaction of epoxides of unsaturated fatty acids by CDs has not been described. In contrast, some studies dealing with the opening reaction of fatty epoxides by mono or disaccharides have been reported. For instance, Queneau et al. [31,32] studied the production of hydroxyethers of sucrose, trehalose, and isomalt in basic medium with 1,2-epoxidodecane. Albarrán-Preza et al. [33] have also carried out the reaction between epoxidized linseed oil and xylitol in the presence of ZnCl_2_, in *N*-methyl-2-pyrrolidone (NMP). On the other hand, in our case, the epoxide group is located in the middle of a fatty chain, which may imply a steric hindrance. In addition, this product also has an ester group, which could react preferentially through transesterification depending on the conditions used.

The opening reaction of methyl oleate epoxide by CDs was first studied in solution in a basic medium. In the same way as Manta et al. [29], we used aqueous NaOH solution (0.6 M) for the reaction between β-CD and excess of fatty ester epoxide. After 24 h at room temperature or even 48 h at 100 °C, the expected product was not detected and the epoxide was hydrolyzed to give the corresponding diol and polymerization products identified by ESI^+^-MS. The use of Et_3_N or NaH as base in anhydrous DMF also failed. It should be noted that the same reactions conducted with 6^I^-amino-6^I^-deoxy-β-CD (β-CDNH_2_) were unsuccessful too. Tests were then performed with Lewis acids, such as ZnCl_2_, FeCl_3_, and BF_3_-Et_2_O as well as Brønsted acids, such as para-toluenesulfonic acid (APTS) and H_2_SO_4_. All the reactions conducted in anhydrous DMF, at room temperature or 80 °C, during 24 or 48 h, led to the hydrolysis of the epoxide group followed by the polymerization of the methyl oleate epoxide. It should be noted that the microwave conditions (300 W, 80 °C) also failed to lead to the formation of L-CDs from epoxide (from 0.5 to 2.5 eq.) and 1 eq. of β-CD lyophilized catalyzed by H_2_SO_4_ (from 0.05 to 0.25 eq.) in anhydrous DMF. Even after 75 min, we did not see the formation of the expected product.

Finally, we attempted the ring opening of epoxide using ball milling, a mecanosynthesis technique widely used in green chemistry [12,13]. Very recently, Cravotto et al. [14] described ring opening of low-boiling epoxides by CDs under high-energy ball milling conditions and in the presence of NaOH, leading to well-known HP-β-CD, HP-γ-CD and epichlorhydrin cross-linked CD-polymers. We tried to initiate the opening reaction of methyl oleate epoxide by β-CD with various bases and acids in the absence of solvent. For the various tests performed, the β-CD was previously lyophilized, the oleate epoxide/CD ratio was 0.5 to 2.5 and the base or acid amount was 0.1 to 0.5 eq. relative to the β-CD. Initially, we tested the reaction with triethylamine and KOH as strong mineral base. As with the result obtained in solution, the expected product was not observed nor the polymerized by-products and hydrolyzed epoxide. However, the same reaction performed with APTS led to expected lipidyl-β-CD with a weak yield of 10%, as confirmed by the ESI^+^-MS analysis showing a signal corresponding to the [M + Na]^+^ ion of the desired product at *m*/*z* 1469.62 (SI, Figure S14).

**Scheme 2:**
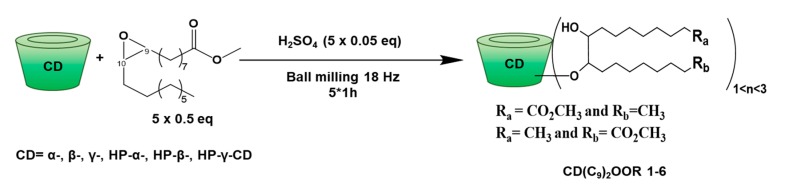
Opening reaction of methyl oleate epoxide by CDs using ball milling.

New attempts in the presence of a stronger acid such as H_2_SO_4_ have been successfully made. To summarize, the optimized experimental conditions are the following: 0.5 eq. of methyl oleate epoxide and 0.05 eq. of H_2_SO_4_ are mixed previously for 10 min and then added to 1 eq. of β-CD freeze-dried with a 1-h grinding cycle (Scheme 2).

The process is repeated for five cycles to avoid the formation of a sticky paste harmful for reactivity [34,35,36], leading to a final oleate epoxide/CD ratio of 2.5 in the presence of 0.25 eq. of H_2_SO_4_. It should be noted that the completion of a sixth one-hour cycle did not result in an improvement in performance.

**Figure 1:**
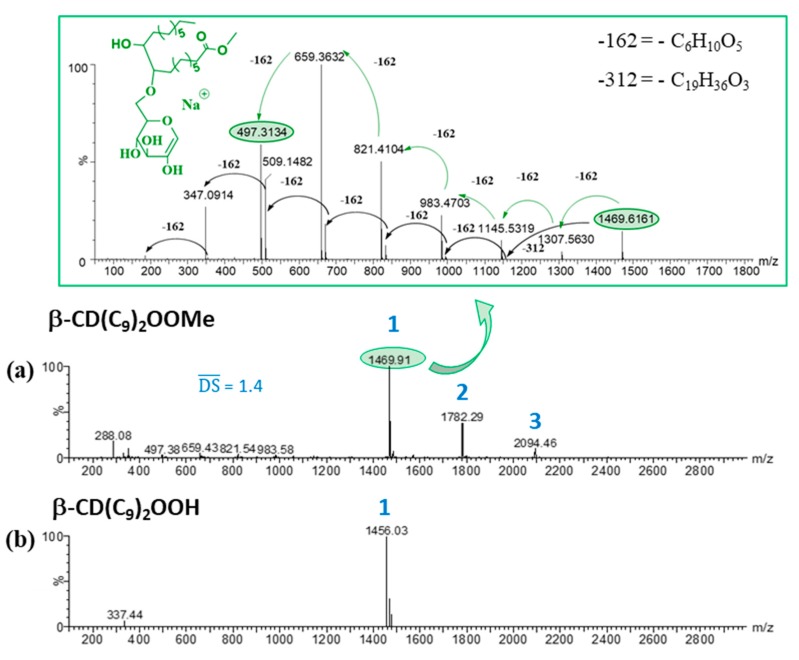
Electro spray ionization mass spectrometry (ESI^+^-MS) spectra of β-CD(C_9_)_2_OOMe **1** (**a**) and β-CD(C_9_)_2_OOH 8 (**b**). For 1 the MS/MS spectrum of the mono grafted species (*m/z* 1469.63) is shown. The average DS value was calculated using the following equation: $\bar{\mathrm{DS}}=\sum_{i}{\mathrm{DS}}_{i}{\times I}_{i}/\sum_{i}I_{i}$

The reaction mixture retained its powder state and the resulting β-CD(C_9_)_2_OOMe **1** exhibited between 1 and 3 substituents as displayed on Scheme 2. This crude reaction mixture was analyzed by ESI^+^-MS (SI, Figure S14) revealing the expected grafted derivatives of DS = 1, 2, and 3 and also free β-CD, the starting epoxide and its polymerized co-products. According to MS data, we were able to determine the average degree of substitution $\bar{\mathrm{DS}}$ of our L-CDs mixture using the respective intensity of each species. This result allows the calculation of an average molar mass (MWa) and to access the reaction yield. The $\bar{\mathrm{DS}}$ calculated from ESI^+^-MS spectrum was 1.4 and the yield achieved was 55% after treatment allowing an efficient elimination of the starting materials without modification of the grafting distribution profile (Figure 1, Table 1). In addition, for β-CD(C_9_)_2_OOMe **1**, the effective grafting of 1, 2, or 3 methyl oleate chains was demonstrated by MS/MS experiments on their respective [M + Na]^+^ ions at *m*/*z* 1469.63, 1781.89, and 2094.16. For illustration (Figure 1), the MS/MS spectrum obtained for the mono-grafted species highlighted two fragmentation pathways. On the one hand, six successive losses of glucopyranose unit (−162 Da) were observed [19] leading to the characteristic fragment ion at *m*/*z* 497.31, which was attributed to a sodiated mono grafted dehydro glucose unit. On the other hand, a loss of 312 Da corresponding to the methyl oleate chain occurred first and was then followed by six successive losses of 162 Da. This result is a proof of the effective methyl oleate epoxide opening by the β-CD. The MS/MS spectra of DS = 2 and 3 are in accordance with the grafting of 2 and 3 lipidyl chains without, as for DS = 1, any assumption of the regioselectivity (SI, Figure S15).

In order to obtain a family of CD(C_9_)_2_OOMe, the ball milling protocol optimized for the native β-CD was transposed to the main derivatives of commercial β-CD: RAMEB and HP-β-CD, previously lyophilized. With RAMEB, the expected reaction did not occur while HP-β-CD gave the desired product **5** with 35% yield and an average DS of 1.3 (SI, Figure S16). This difference in reactivity can be explained, on the one hand, by the presence of hydroxyl groups of the hydroxypropyl substituents of HP-β-CD as reactive, or even more accessible, than the hydroxyl groups of the glucoses composing the β-CD. On the other hand, in the case of RAMEB, the substitution of hydroxyl groups by methyl groups reduces the accessibility of those remaining, making this CD less reactive compared to native β-CD and HP-β-CD. We successfully transposed this optimized reaction and purification conditions to the native α-CD and γ-CD as well as the HP-α-CD and HP-γ-CD derivatives to obtain the L-CDs family. All the results obtained are presented in Table 1.

The $\bar{\mathrm{DS}}$ calculated from ESI^−^-MS are also presented, and are in agreement with the $\bar{\mathrm{DS}}$ obtained with ESI^+^-MS. Thus, we obtained six bicatenary compounds that differ by the nature of the CD used but with homogeneous average $\bar{\mathrm{DS}}$ between 1.1 and 1.4, according to the positive or negative ionization mode of the ESI-MS analyses. The yields obtained range from 28 to 55% after purification, which remains satisfactory for modified CDs obtained in a single step. It should be noted that better results are observed with the β-CD, probably because of a greater affinity between this CD and the oleate epoxide. This, combined with reasonable reaction times (5 × 1 h) and with the absence of solvent, allowed the compounds to be obtained on a gram scale in the laboratory. This easy access as well as effective purification conditions allow an evaluation of their properties. In order to better understand the reaction, methyl α-d-glucopyranoside, considered as a unit constituting CDs, has been used as the raw material. However, in the same experimental conditions, we obtained a pasty and sticky medium that inhibited the reaction. The addition of SiO_2_ as a milling aid [37] did not lead to the opening reaction of methyl oleate epoxide despite several tests. These results seem to be in agreement with an interaction of the fatty chain with the CDs in solid phase, promoting the opening of the epoxide by the latter.

**Scheme 3:**
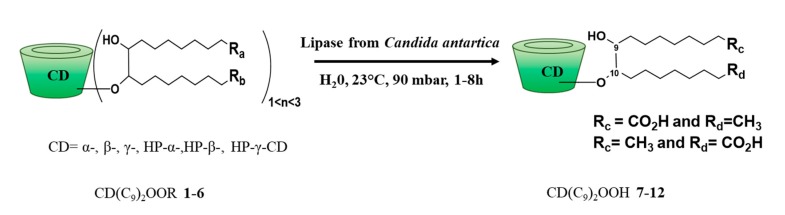
Enzymatic hydrolysis reaction.

Finally, the enzymatic hydrolysis on the terminal ester of the CD(C_9_)_2_OOMe fatty chain was carried out with immobilized lipase from *Candida antarctica* to produce the corresponding CD(C_9_)_2_OOH (**7–12**) with very good yield at 100 mg scale (Scheme 3, Table 2). The products were isolated by simple filtration of the immobilized lipase followed by freeze-drying to obtain the derivative CD(C_9_)_2_OOH.

As an illustration, the total conversion of β-CD(C_9_)_2_OOMe by the enzymatic hydrolysis reaction was evidenced by its ESI^+^-MS spectrum (Figure 1). Only the compound of DS = 1 was recovered. In addition, ^13^C NMR analysis of β-CD(C_9_)_2_OOH **8** allowed us to detect the disappearance of methyl ester signals (51.7 ppm methyl signal and 174.4 ppm terminal ester carbonyl signal) and the presence of 176.5 ppm acid group carbonyl signal (SI, Figure S17). The enzymatic hydrolysis reaction was faster for some derivatives, such as γ-CD(C_9_)_2_OOMe **3** and HP-CD(C_9_)_2_OOMe **4**–**6**. As for β-CD(C_9_)_2_OOH **8,** all CD(C_9_)_2_OOH derivatives had only one grafted substituent probably because of the experimental isolation conditions since the final filtration in water favored the most hydrophilic compounds.

### 3.2. Characterization

**Scheme 4:**
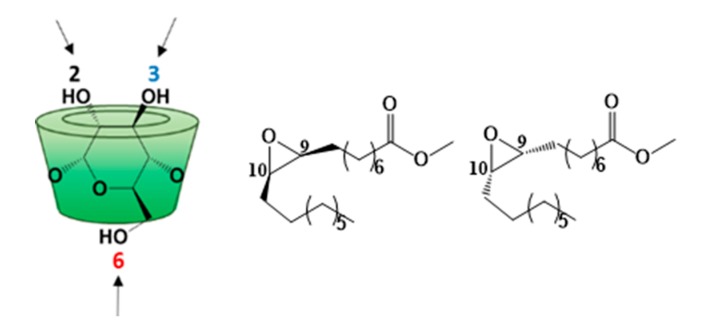
Structures of cyclodextrins (CDs) and methyl oleate epoxide.

The methylated L-CD derivatives **1**–**6** are made up of CDs carrying between 1 to 3 fatty chains (Figure 1), each of these components may have several isomers. The objective of this part is to carry out a further characterization of these complex mixtures in order to highlight the presence of isomers because of a potential regioselectivity and/or stereoselectivity of the epoxide opening reaction. Indeed, this reaction could involve the different hydroxyl groups of the CDs (2, 3, and 6) as well as the carbon C_9_ and C_10_ of the epoxide (Scheme 4).

**Figure 2:**
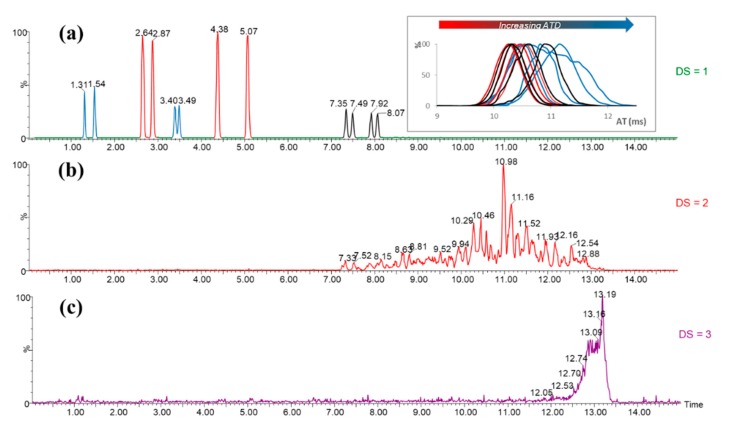
[M + Na]^+^ extracted ion chromatograms (EICs) of **1**: DS = 1 *m*/*z* 1469.63 (**a**), DS = 2 *m*/*z* 1781.89 (**b**), and DS = 3 *m*/*z* 2094.16 (**c**). For DS = 1, the arrival time distributions (ATDs) of the 12 isomers obtained using a travelling wave mobility cell (TWIMS) are presented.

To illustrate the structural characterization of these compounds, we focused on the β-CD(C_9_)_2_OOMe derivative **1** as an example to describe our multi-technique approach. First, 1D and 2D NMR experiments were implemented. However, the ^1^H NMR spectra proved to be extremely complicated because of a significant overlap in the frequencies of the proton signals because of the loss of symmetry of the CD cavity and its variable substitution rate. Similarly, ^13^C NMR spectra have many carbon signals whose chemical shifts are too similar, making it difficult to fully attribute them (SI, Figure S18). Only 2D experiments (COSY, HSQC, and HMBC) allowed a partial assignment (SI, Figure S19). This mixture was then analyzed by liquid chromatography coupled to mass spectrometry (LC/MS) using a C_8_ column for the separation. The extracted ion chromatograms (EICs) corresponding to [M + Na]^+^ ions of DS = 1 (*m*/*z* 1469.63), DS = 2 (*m*/*z* 1781.89), and DS = 3 (*m*/*z* 2094.16) species are depicted in Figure 2. In the case of DS = 1, the 12 chromatographic peaks can be explained by the 12 isomers that can be formed during the epoxide opening reaction. They correspond to the respective contribution of the three OH groups of β-CD, the two carbon of epoxide ring and the two configurations available for this ring (Scheme 4 and SI, Figure S20). For DS = 2 and DS = 3 (Figure 2b,c), the significant increase in the number of possible isomers has led to more complex chromatograms with a separation reaching a limit with a broad peak observed for DS = 3.

**Figure 3:**
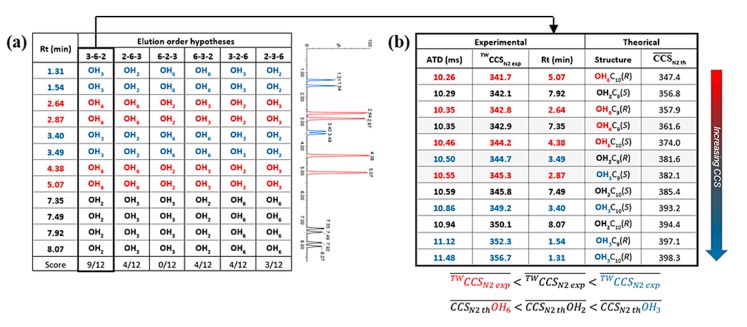
(**a**) Assignment hypotheses of the β-CD hydroxyl groups (OH_2_, OH_3_, and OH_6_) involved in the epoxide opening reaction according to LC/MS elution and MS/MS groups. (**b**) Comparison of the increasing ranking of $\bar{\mathrm{CCS}}$_N2 th_ with those of ^TW^CCS_N2 exp_ in the case of assignment hypothesis (3-6-2). The highlighted grey lines correspond to the three negative matches between expected OH assignment hypothesis and CCS prediction, leading to a score of 9/12.

In order to distinguish the 12 isomers of **1** DS = 1, a MS/MS acquisition was performed on each chromatographic peak. All the 12 precursor ions ([M + Na]^+^, *m*/*z* 1469.63) gave fragments with the same *m*/*z*. However, the comparison of their relative intensities has made it possible to form three groups which were assumed to be regioisomer groups (blue, red and black, Figure 2a and SI, Figure S21) without the possibility of OH position assignment. Thus MS/MS constitutes the first experiment discriminating isomers. The next step was to study the benefit of ion mobility (IMS, ion mobility spectrometry) to complete the structural characterization. IMS is an analytical technique that separates ions in the gas phase according to their size, shape, and charge. This technique hyphenated with a mass spectrometer (IMS-MS) allows the separation of isobaric compounds depending on their structure. Indeed, the arrival time distribution (ATD) of each ion crossing the mobility cell can be correlated to its collision cross section (CCS, Ω in Å^2^) which is an intrinsic physicochemical characteristic corresponding to the surface of interaction between the ion and the gas molecules (N_2_) filling the mobility cell [38]. First, the ATD of each isomer was recorded. The ion mobility spectrum [39] revealed a partial separation of the different isomers with the blue group being the most delayed (Figure 2a). The experimental CCS (^TW^CCS_N2 exp_) of each species was deducted from the arrival time distributions (ATDs) by external calibration. Second, 3D structures of DS = 1 isomers were simulated with minimal internal energy and their theoretical CCS (CCS_N2 th_) were predicted according to three methods: approximation projection (PA), exact hard spheres scattering (EHSS), and trajectory method with Lennard-Jones parameters (TMLJ) [40]. The comparison of their $\bar{\mathrm{CCS}}$_N2 th_ increasing ranking with the ^TW^CCS_N2 exp_ made it possible to propose a regioisomer assignment according to the CD hydroxyl position involved in the reaction. Based on the results obtained on the one hand by LC/MS (elution of 12 isomers, Figure 2a) and on the other hand by MS/MS (3 distinct groups of 4 isomers each, Figure 2a and Figure S21), a set of six different hypotheses of elution order was obtained corresponding to the six columns of Figure 3a. For each column, we proposed a correlation between the predicted assignment of OH_2_, OH_3_, and OH_6_ and the experimental vs. theoretical CCS (^TW^CCS_N2 exp_ vs. $\bar{\mathrm{CCS}}$_N2 th_), which led to a score (equivalent to the number of positive matches). The best correlation (9/12) was obtained for the elution order 3-6-2 (Figure 3b) and corresponds to the following hypothesis: OH_3_ (blue group), OH_6_ (red group), and OH_2_ (black group). The assignment of the most intense peaks (red) to OH_6_ position is in agreement with a greater reactivity of primary OH in our conditions. Moreover, the IMS attribution of the OH_2_ position, the only glucopyranose hydroxyl group oriented toward the inside of the CD cavity, seems to be in agreement with its MS/MS profile which was found to be very different from those of the other OH positions (SI, Figure S21). In particular, lower relative intensities of the fragment ions at *m*/*z* < 600 have been observed for the OH_2_ (black group), indicating a probable higher stability of the L-CD structure in this case. This innovative approach seems to be a promising tool to elucidate complex isomer mixtures such as cyclodextrin derivatives.

Similarly, L-CDs synthesized from α-CD and γ-CD were analyzed by LC/MS and the presence of 12 isomers for DS = 1 species was also observed (SI, Figure S22). It should be pointed out that after enzymatic hydrolysis, the saponified DS = 1 compounds kept the same number of isomers than the esterified ones (SI, Figure S23).

### 3.3. Self-Organization Properties

**Figure 4:**
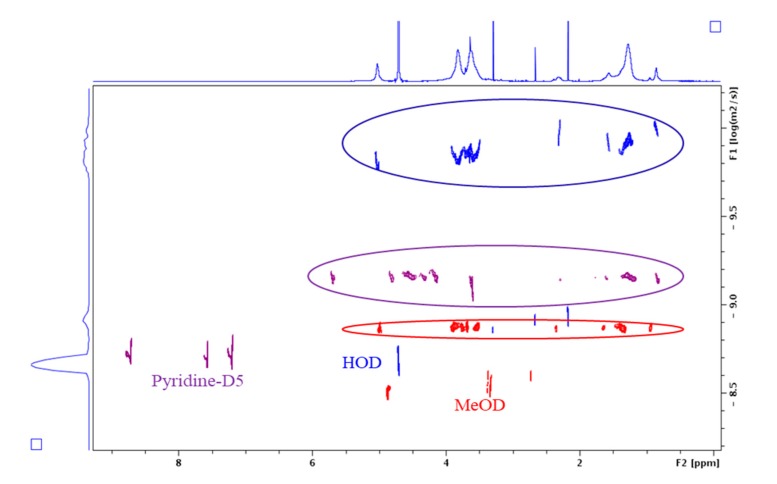
Overlapping DOSY maps obtained for **1** in D_2_O (blue spots), pyridine-D_5_ (purple spots), and MeOD (red spots) (12 mM, 600 MHz, 298 K).

Samples of **1** were analyzed by DOSY in different solvents, deuterium oxide, MeOD, and pyridine-d_5_ (Figure 4). This two-dimensional NMR experiment can discriminate molecules or objects according to their diffusion coefficient D directly linked to the translational motion of molecules in solution and whose value depends on the size of the object [41]. The bigger the molecule or object is, smaller the D value will be. It should be noted that the D value of 1 varies with the solvent, and water gives the lowest value with D = 1.65 × 10^−10^ m^2^/s, compared to D = 7.23 × 10^−10^ m^2^/s and 1.34 × 10^−9^ m^2^/s in pyridine-d_5_ and MeOD, respectively. This could suggest a potential phenomenon of aggregation of β-CD(C_9_)_2_OOMe **1** in aqueous medium.

**Figure 5:**
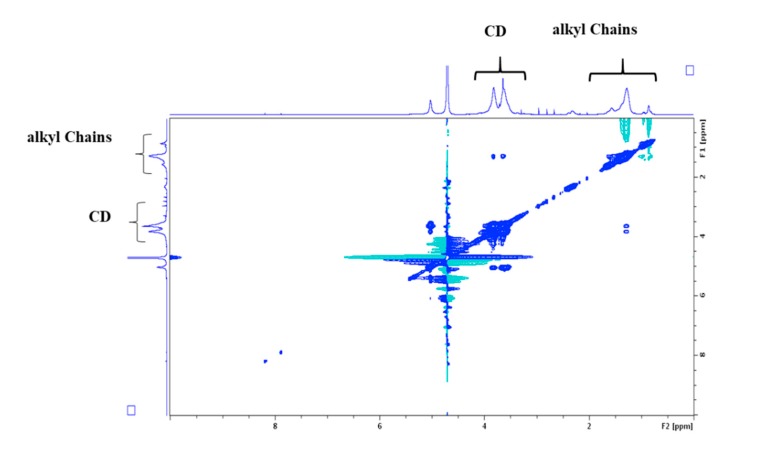
Partial contour plot of a T-ROESY experiment (spin lock: 300 ms, 22 dB) performed at 600 MHz on **1** at 12 mM in D_2_O.

In order to study the dipolar interactions between protons of the CD cavity and those of the fatty chains, a T-ROESY NMR experiment was performed on β-CD(C_9_)_2_OOMe **1** in D_2_O (Figure 5) [42]. Strong cross-correlation peaks between alkyl protons and protons of CD moiety have been observed revealing interactions of the grafted chains with the CD cavity. Two types of interactions can explain this result: an intramolecular interaction between a fatty chain and the cavity of its own CD (self-inclusion) or an intermolecular interaction between a chain and the cavity of another CD. The latter is more compatible with the observed self-aggregation phenomenon.

In addition, the self-assembling properties of **1** were evaluated by DLS in D_2_O. For this purpose, nanoparticles were prepared according to a method based on the hydration of a lipid film [43]. In summary, **1** was solubilized in methanol, then the solvent was evaporated under nitrogen stream to obtain a film, dried under vacuum. The water was added, and the sample was sonicated. DLS analysis of a 25 µM solution of **1** confirmed the presence of nanoparticles with an average hydrodynamic diameter of 145 nm (PDI = 11%). Its CAC value was also estimated between 5 and 8 µM by the DLS using a series of samples with concentrations ranging from 0.5 µM to 20 µM while its solubility in water is assessed at 12 mM.

### 3.4. Preliminary Studies for L-CD Vectorization Potential Use of In Vitro Blood-Brain and Intestinal Barriers Models

**Figure 6:**
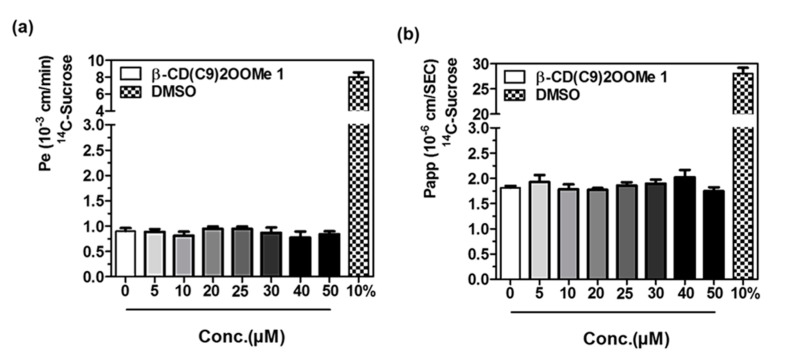
^14^C-Sucrose permeability (Pe) and apparent permeability (Papp) assessment in two in vitro models treated with different concentrations of **1**. DMSO 10% was used as the positive control of biological barrier disruptions. Bars represent the mean of six filters + standard error of the mean. (**a**) represents the Pe of ^14^C-Sucrose in the brain-like endothelial cells (BLECs) model and (**b**) represents the Papp of ^14^C-sucrose in the intestinal Caco-2 model.

As part of their use as vectors of active ingredients, selected lipidyl cyclodextrins effect on barrier integrity was then assessed using the two human in vitro models of physiological barriers, i.e., intestinal and blood-brain barriers were widely used to investigate molecules and xenobiotics toxicities and permeabilities [24,44,45]. Permeability of small integrity marker, ^14^C-sucrose, across the monolayer of cells was measured in the presence of 5, 10, 20, 25, 30, 40, and 50 µM of cyclodextrin or 10% DMSO used as positive control of biological barrier disruptions. As shown in Figure 6, permeabilities of ^14^C-sucrose are not increased by the different tested concentrations of L-CD **1** suggesting that this molecule does not show any deleterious effect on these barriers. Indeed, in the tested conditions, β-CD(C_9_)_2_OOMe **1** does not alter the integrity of the BBB (Pe < 1.5 10^−3^ cm/min) or the intestinal barrier (Papp < 5 10^−6^ cm/s). Note that the same studies performed on the γ-CD analog 3 also demonstrated the absence of toxicity (Figure S24). Interestingly, these results open access to deeper biological research to evaluate their vectorization potential.

## 4. Conclusions

New L-CDs derivatives were synthesized using a fatty ester epoxide by means of alternative methods (solvent-free techniques, use of enzymes). The ring opening of methyl oleate epoxide using ball milling led to easy preparation of bicatenary amphiphilic CDs of low DS. Furthermore, enzymatic hydrolysis of the terminal ester gave the corresponding carboxylic acid analogs with very good yields and allowing a modulation of the hydrophilicity of the compounds. In our conditions, the coupled reactions were carried out on the main free CDs (α-, β-, and γ-CDs) or on the hydroxypropylated analogues. We are planning to evaluate other derivatives in subsequent studies as well. Despite the low and controlled DS, the final products were obtained in the form of complex mixtures requiring an extensive structural analysis using mainly mass spectrometry and NMR spectroscopy. The powerful IMS-MS technique has been demonstrated in this work to be an effective tool for a better understanding of complex isomer mixtures. Indeed, it was possible to accurately assign the CD hydroxyl position (2, 3, 6) involved in the epoxide opening reaction which was not directly possible only based on the relative intensity differences observed on the MS / MS spectra. These preliminary IMS-MS data also opened access to additional structural information concerning the regioselectivity and stereoselectivity of the epoxide carbon atoms (C_9_, C_10_). In order to validate this approach, further investigations are currently underway.

The self-assembling properties of **1** were evaluated in the D_2_O showing the presence of stable nanoparticles related to a low CAC value. In addition, as part of their use as vectors of active ingredients, these products were submitted to an integrity study on an in vitro model of the blood-brain-barrier (i.e., the BLEC model) and the intestinal epithelium (Caco-2 model). No toxicity has been demonstrated, suggesting that applications for the vectorization of active ingredients or drugs can be expected.

## Figures and Tables

**Table 1:** CD(C_9_)_2_OOMe derivatives **1**–**6** obtained by ball milling (the experimental conditions are depicted Scheme 2).

CD(C_9_)_2_OOMe	$\bar{\mathrm{DS}}$ (ESI)^+^	$\bar{\mathrm{DS}}$ (ESI)^−^	MWa g.mol^−1^	Yield %
β-CD(C_9_)_2_OOMe 1	1.4	1.3	1577.3	55%
α-CD(C_9_)_2_OOMe 2	1.3	1.2	1378.2	28%
γ-CD(C_9_)_2_OOMe 3	1.3	1.2	1712.5	33%
HP-α-CD(C_9_)_2_OOMe 4	1.2	1.1	1571.7	33%
HP-β-CD(C_9_)_2_OOMe 5	1.3	1.3	1892.5	35%
HP-γ-CD(C_9_)_2_OOMe 6	1.2	1.2	2031.9	44%

**Table 2:** CD(C_9_)_2_OOH derivatives **7**–**12** obtained by enzymatic hydrolysis (the experimental conditions are depicted on Scheme 3).

CD(C_9_)_2_OOH	Time (h)	MWa (g.mol^−1^)	Yield
α-CD(C_9_)_2_OOH **7**	4.5	1271.0	78%
β-CD(C_9_)_2_OOH **8**	8	1433.0	69%
γ-CD(C_9_)_2_OOH **9**	1.5	1595.0	89%
HP-α-CD(C_9_)_2_OOH **10**	2	1499.5	82%
HP-β-CD(C_9_)_2_OOH **11**	2	1736.3	84%
HP-γ-CD(C_9_)_2_OOH **12**	1	1887.3	89%

## Supplementary Materials

The following are available online at https://www.mdpi.com/2218-273X/10/2/339/s1. Figure S1: ESI^+^-HRMS (a), ^1^H NMR (b) and ^13^C NMR spectra (c) of β-CD(C_9_)_2_OOMe **1**. Figure S2: ESI^+^-HRMS (a), ^1^H NMR (b), and ^13^C NMR spectra (c) of α-CD(C_9_)_2_OOMe **2**. Figure S3: ESI^+^-HRMS (a), ^1^H NMR (b), and ^13^C NMR spectra (c) of γ-CD(C_9_)_2_OOMe **3**. Figure S4: ESI^+^-HRMS (a), ^1^H NMR (b), and ^13^C NMR spectra (c) of HP-α-CD(C_9_)_2_OOMe **4**. Figure S5: ESI^+^-HRMS (a), ^1^H NMR (b), and ^13^C NMR spectra (c) of HP-β-CD(C_9_)_2_OOMe **5**. Figure S6: ESI^+^-HRMS (a), ^1^H NMR (b), and ^13^C NMR spectra (c) of HP-γ-CD(C_9_)_2_OOMe **6**. Figure S7: ESI^+^-HRMS (a), ^1^H NMR (b), and ^13^C NMR spectra (c) of α-CD(C_9_)_2_OOH **7**. Figure S8: ESI^+^-HRMS (a), ^1^H NMR (b), and ^13^C NMR spectra (c) of β-CD(C_9_)_2_OOH **8**. Figure S9: ESI^+^-HRMS (a), ^1^H NMR (b), and ^13^C NMR spectra (c) of γ-CD(C_9_)_2_OOH **9**. Figure S10: ESI^+^-HRMS (a), ^1^H NMR (b), and ^13^C NMR spectra (c) of HP-α-CD(C_9_)_2_OOH **10**. Figure S11: ESI^+^-HRMS (a), ^1^H NMR (b), and ^13^C NMR spectra (c) of HP-β-CD(C_9_)_2_OOH **11**. Figure S12: ESI^+^-HRMS (a), ^1^H NMR (b), and ^13^C NMR spectra (c) of HP-γ-CD(C_9_)_2_OOH **12**. Figure S13: ^1^H NMR spectra of methyl oleate (a) and of epoxidated methyl oleate (b). Figure S14: ESI^+^-MS spectrum of the ball milling reaction medium of the methyl oleate epoxide opening with the β-CD assisted by APTS. Figure S15: ESI^+^-MS/MS spectra of the [M + Na]^+^ ions of DS = 2 (a) and DS = 3 (b) species of β-CD(C_9_)_2_OOMe **1** obtained from ball milling. Figure S16: ESI^+^-MS spectrum of the of HP-β-CD(C_9_)_2_OOMe **5** obtained from ball milling, showing mono (green), di (red), and tri (blue) grafted species. Figure S17: ^13^C NMR spectra of **8** (a) and of **1** (b). Figure S18: ^13^C NMR spectra of methyl oleate epoxide (a), **1** (b), and native β-CD (c). Figure S19: 2D NMR spectra of **1**: HSQC (a), COSY (b), and HMBC (c) (600 MHz, pyridine-D5, 298 K). Figure S20: Opening reaction of epoxide by free cyclodextrin. Figure S21: ESI^+^-MS/MS (95 eV) spectra of the twelve DS = 1 isomers ([M + Na]^+^ *m*/*z* 1469.63) of **1** allowing the distinction of three groups of regioisomers. Figure S22: [M − H]^−^ reconstituted ion chromatograms of **2**. Figure S23: [M − H]^−^ reconstituted ion chromatograms of **7**. Figure S24: 14C-Sucrose permeability (Pe) and apparent permeability (Papp) assessment in two in vitro models treated with different concentrations of **3**.
