# Supplementary material for: New Lipidyl-Cyclodextrins Obtained by Ring Opening of Methyl Oleate Epoxide Using Ball Milling
Source: Biomolecules. 2020 Feb 20;10(2):339. doi: 10.3390/biom10020339 (PMC7072689; doi:10.3390/biom10020339)
Supplement: Supplementary file 1 [file biomolecules-10-00339-s001.pdf]

Supplementary materials

New Lipidyl-Cyclodextrins Obtained by Ring Opening of Methyl Oleate Epoxide using Ball Milling

Estefania Oliva[1], David Mathiron[2], Sébastien Rigaud[2], Eric Monflier[3], Emmanuel Sevin[4], Hervé Bricout[3], Sébastien Tilloy[3], Fabien Gosselet[4], Laurence Fenart[4], Véronique Bonnet[1], Serge Pilard[2] and Florence Djedaini-Pilard[1*]

1 LG2A UMR CNRS 7478, Université de Picardie Jules Verne, 80039 Amiens Cedex, France ; florence.pilard@u-picardie.fr
2 Plateforme Analytique, Université de Picardie Jules Verne, 80039 Amiens Cedex, France ; serge.pilard@u-picardie.fr
3 Univ. Artois, CNRS, Centrale Lille, ENSCL, Univ. Lille, UMR 8181 – UCCS – Unité de Catalyse et Chimie du Solide, F-62300 Lens, France ; eric.monflier@univ-artois.fr
4 LBHE EA 2465, Université d'Artois, rue Jean Souvraz, 62307 Lens Cedex France ; laurence.tilloy@univ-artois.fr

β-CD(C9)2OOMe, **1**

$\overline{DS}$: 1.4
MWa: 1577.3 g/mol
Yield: 55%

[M+Na]$^+$	DS$_1$	DS$_2$
Formula	$C_{61}H_{106}O_{38}Na$	$C_{80}H_{142}O_{41}Na$
Mass	1469.6233	1781.8888
Calc. mass	1469.6260	1781.8924
Error (ppm)	1.8	2.0

(a)

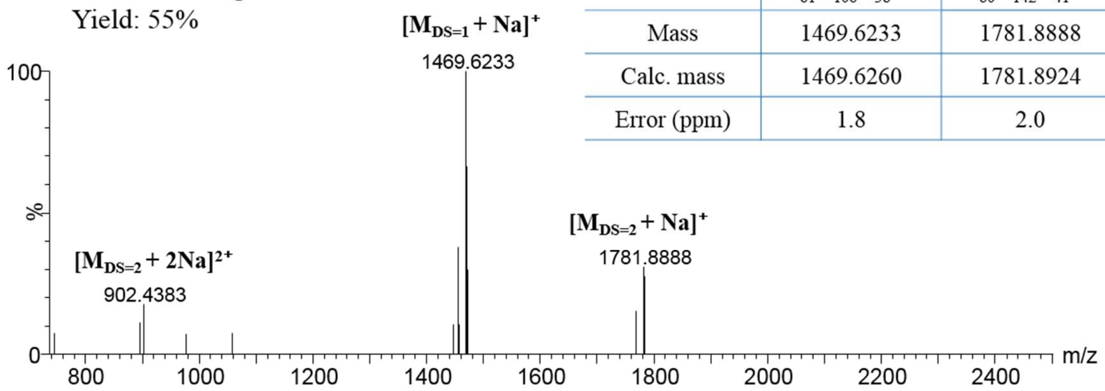

(b)

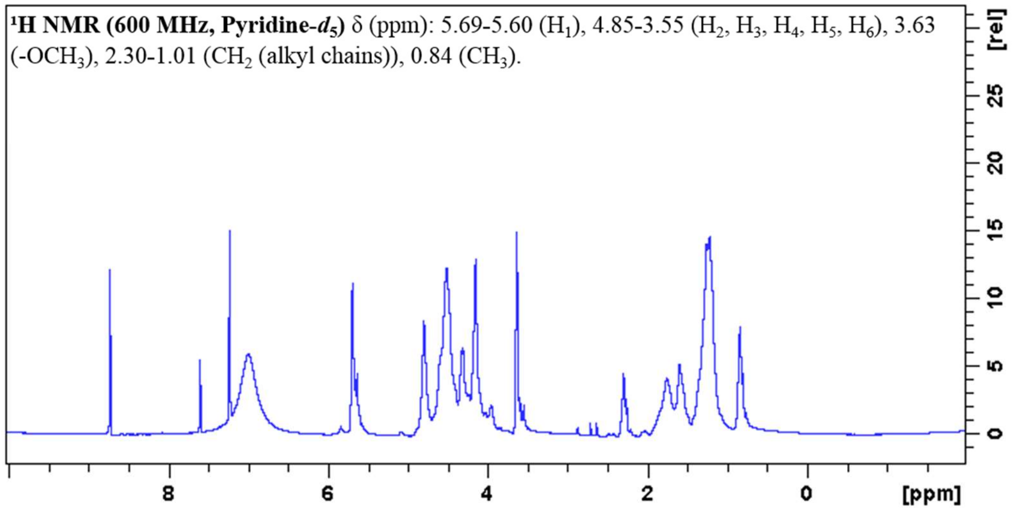

^{1}H NMR (600 MHz, Pyridine-*d*$_5$) δ (ppm): 5.69-5.60 (H_1), 4.85-3.55 (H_2, H_3, H_4, H_5, H_6), 3.63 (-OCH_3), 2.30-1.01 (CH_2 (alkyl chains)), 0.84 (CH_3).

(c) **^{13}C NMR (151 MHz, Pyridine-*d*$_5$)** δ (ppm): 174.4 (C=O), 104.3 (C_1), 85.2-83.0 (C_4), 75.3-72.7 (C_2, C_3, C_5), 62.4-61.6 (C_6), 51.7 (-OCH_3), 34.5-23.0 (CH_2 (alkyl chains)), 14.6 (CH_3).

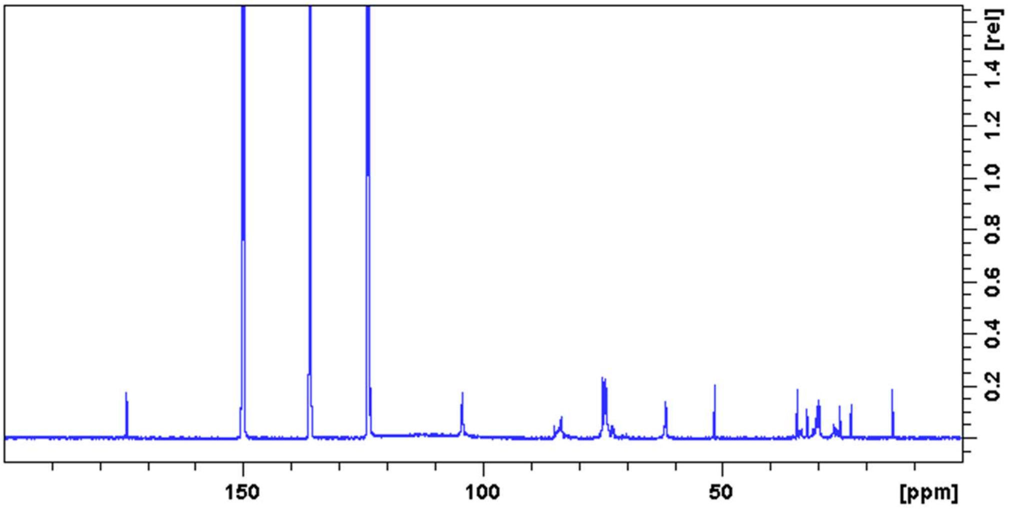

Figure S1: ESI$^+$-HRMS **(a)**, ^{1}H NMR **(b)** and ^{13}C NMR spectra **(c)** of β-CD(C9)2OOMe **1**.

α-CD(C$_9$)$_2$OOMe, **2**

(a)

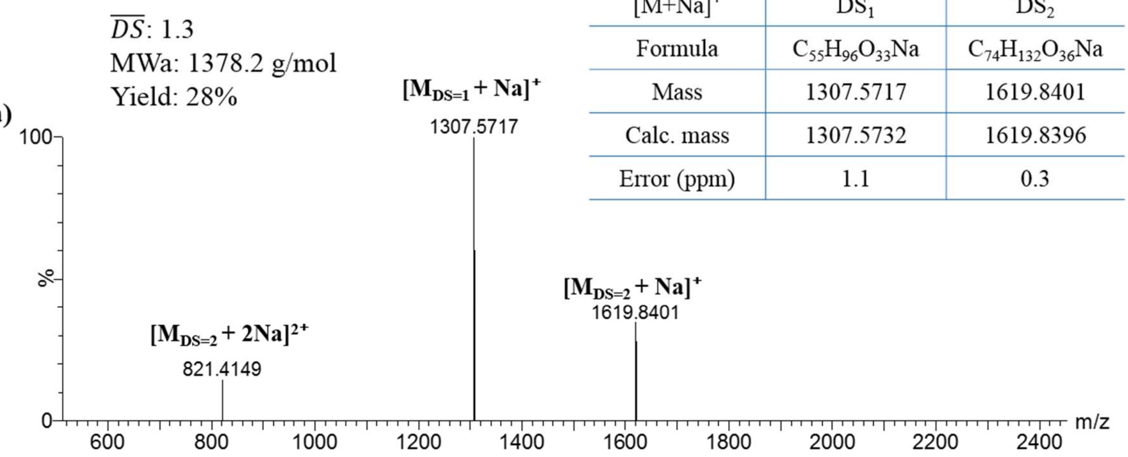

[M+Na]$^+$	DS$_1$	DS$_2$
Formula	$C_{55}H_{96}O_{33}Na$	$C_{74}H_{132}O_{36}Na$
Mass	1307.5717	1619.8401
Calc. mass	1307.5732	1619.8396
Error (ppm)	1.1	0.3

(b)

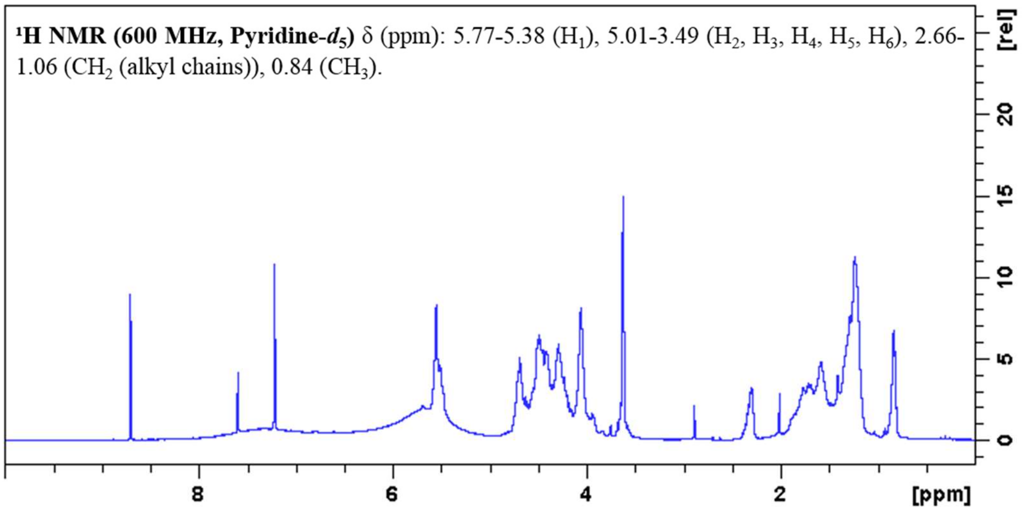

(c) **^{13}C NMR (151 MHz, Pyridine-d_5)** δ (ppm): 174.3 (C=O), 104.5-103.5 (C$_1$), 86.1-82.4 (C$_4$), 76.4-70.0 (C$_2$, C$_3$, C$_5$), 62.4-60.8 (C$_6$), 51.6 (-OCH$_3$), 35.1-22.7 (CH$_2$ (alkyl chains)), 14.6 (CH$_3$).

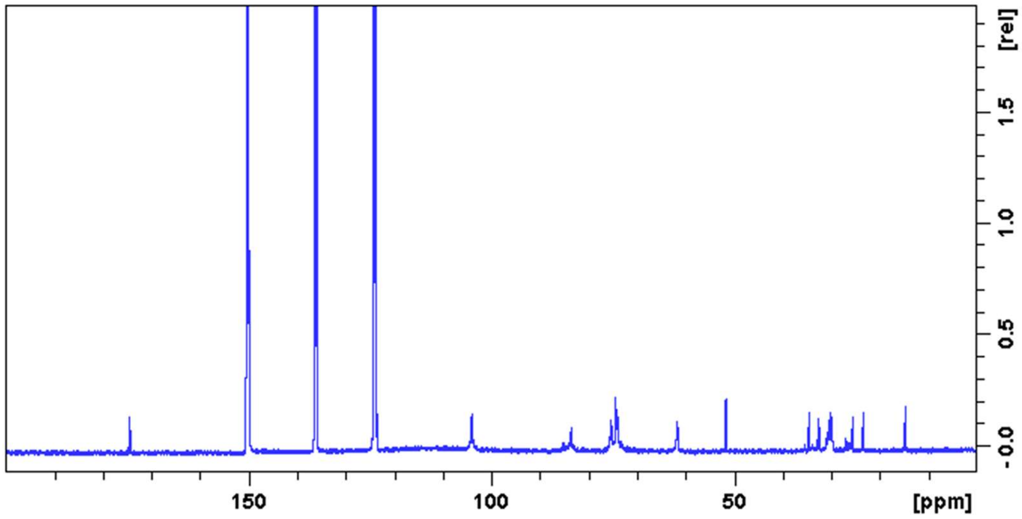

Figure S2: ESI$^+$-HRMS **(a)**, ^{1}H NMR **(b)** and ^{13}C NMR spectra **(c)** of α-CD(C$_9$)$_2$OOMe **2**.

γ-CD(C9)2OOMe, **3**

$\overline{DS}$: 1.3
MWa: 1712.5 g/mol
Yield: 33%

(a)

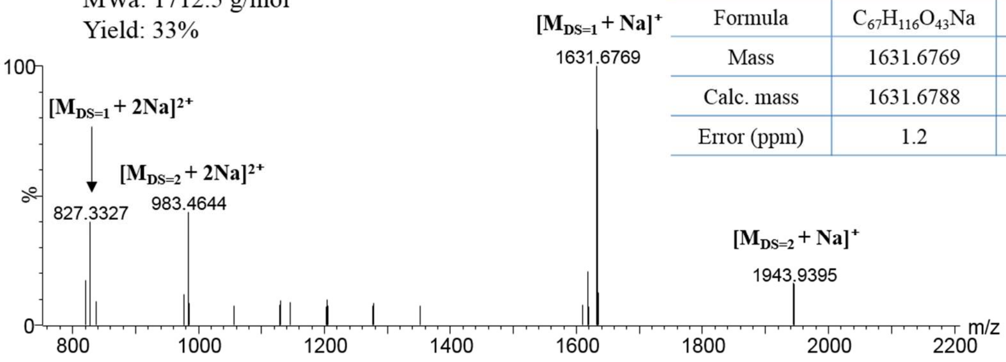

$[M+Na]^+$	DS_1	DS_2
Formula	$C_{67}H_{116}O_{43}Na$	$C_{86}H_{152}O_{46}Na$
Mass	1631.6769	1943.9395
Calc. mass	1631.6788	1943.9452
Error (ppm)	1.2	2.9

(b)

^{1}H NMR (600 MHz, Pyridine-*d*$_5$) δ (ppm): 5.98-5.45 (H_1), 4.79-3.52 (H_2, H_3, H_4, H_5, H_6), 3.63 ($-OCH_3$), 2.50-1.06 (CH_2 (alkyl chains)), 0.81 (CH_3).

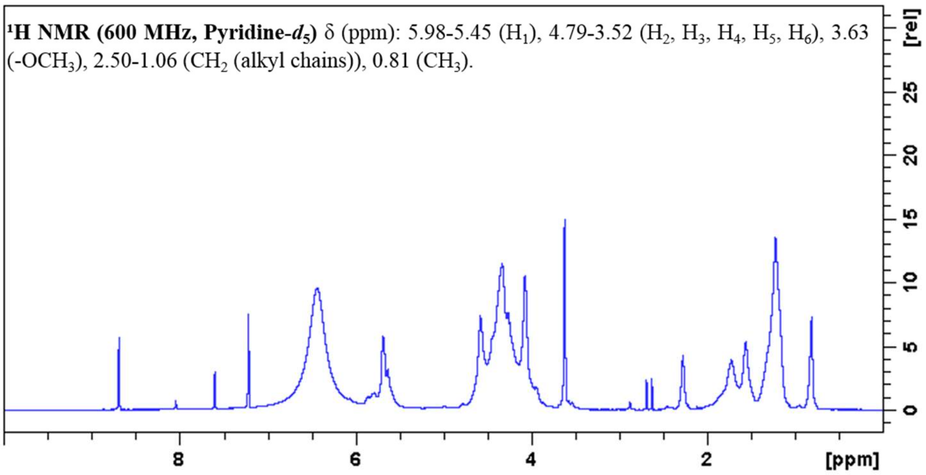

(c) **^{13}C NMR (151 MHz, Pyridine-*d*$_5$)** δ (ppm): 174.2 (C=O), 103.6 (C_1), 85.5-81.0 (C_4), 75.6-71.3 (C_2, C_3, C_5), 63.2-60.9 (C_6), 51.6 ($-OCH_3$), 35.0-22.6 (CH_2 (alkyl chains)), 14.6 (CH_3).

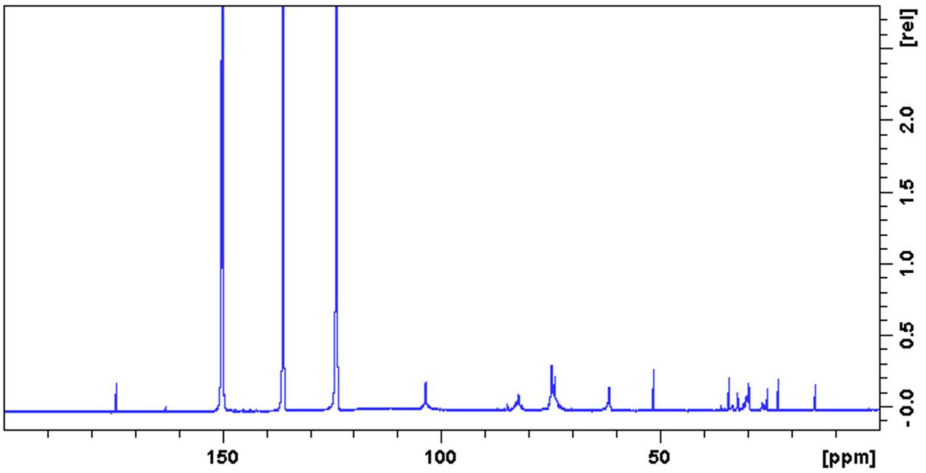

Figure S3: ESI$^+$-HRMS **(a)**, ^{1}H NMR **(b)** and ^{13}C NMR spectra **(c)** of γ-CD(C9)2OOMe **3**.

HP-α-CD(C_9)$_2$OOMe, **4**

$\overline{DS}$: 1.2
MWa: 1571.7 g/mol
Yield: 33%

$[M+Na]^+$	DS_1	DS_2
Formula	$C_{70}H_{126}O_{38}Na$	$C_{86}H_{156}O_{40}Na$
Mass	1597.7808	1852.0050
Calc. mass	1597.7825	1852.0071
Error (ppm)	1.1	1.1

(a)

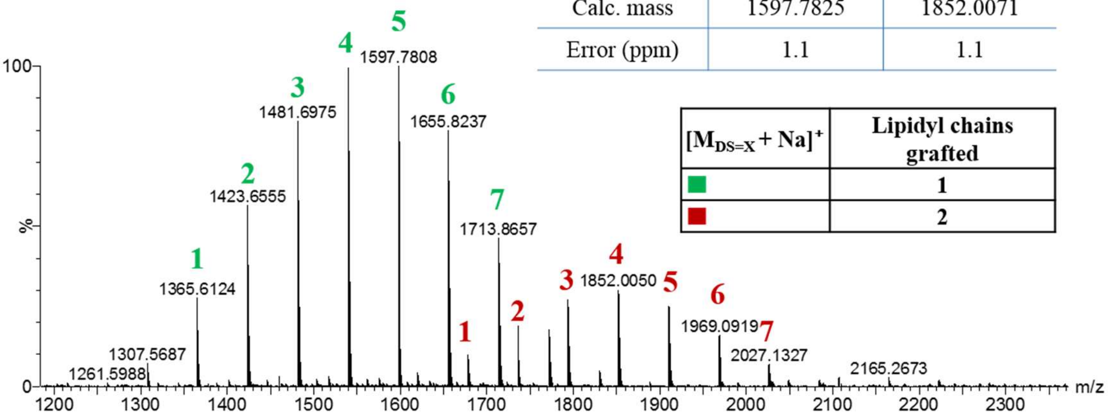

$[M_{DS=X} + Na]^+$	Lipidyl chains grafted
■ (green)	1
■ (red)	2

(b)

^{1}H NMR (600 MHz, Pyridine-d_5) δ (ppm): 6.42-5.24 (H_1), 4.92-3.55 (H_2, H_3, H_4, H_5, H_6), 3.63 (-OCH_3), 2.43-1.46 (CH_2 (alkyl chains)), 1.50-1.03 (CH_3 (hydroxypropyl group)), 0.84 (CH_3 (alkyl chains)).

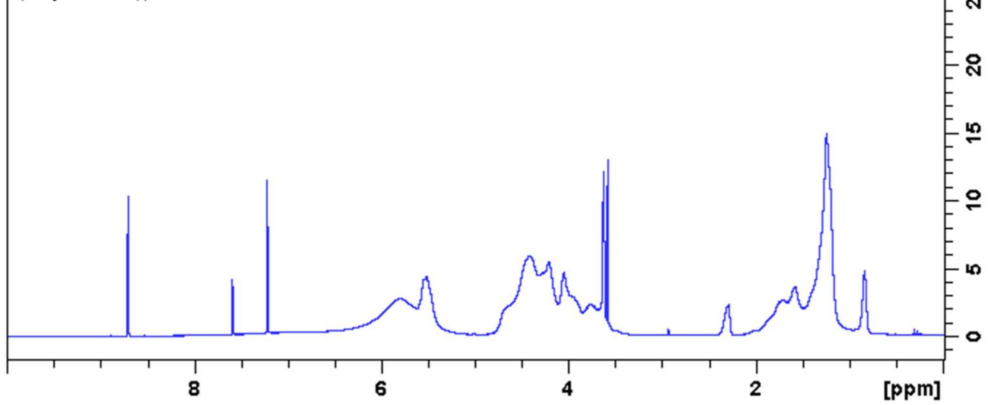

(c)

^{13}C NMR (151 MHz, Pyridine-d_5) δ (ppm): 174.3 (C=O), 105.0-100.5 (C_1), 86.1-77.4 (C_4), 76.0-66.0 (C_2, C_3, C_5), 63.0-60.7 (C_6), 51.6 (-OCH_3), 34.8-22.9 (CH_2 (alkyl chains)), 21.0-19.9 (CH_3 (hydroxypropyl group)), 14.6 (CH_3).

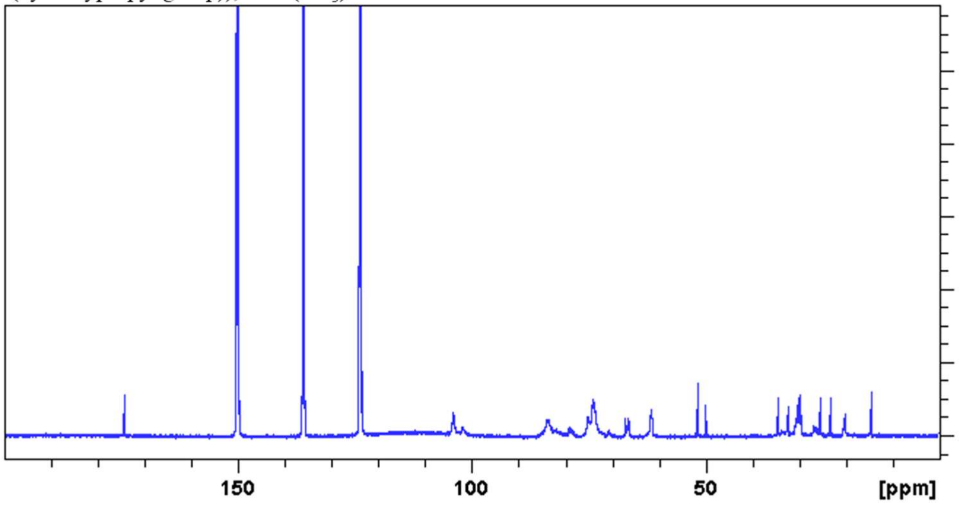

Figure S4: ESI$^+$-HRMS **(a)**, ^{1}H NMR **(b)** and ^{13}C NMR spectra **(c)** of HP-α-CD(C_9)$_2$OOMe **4**.

HP-β-CD(C_9)$_2$OOMe, **5**

$\overline{DS}$: 1.3

MWa: 1892.5 g/mol

Yield: 35%

$[M+Na]^+$	DS_1	DS_2	DS_3
Formula	$C_{82}H_{148}O_{45}Na$	$C_{101}H_{184}O_{48}Na$	$C_{120}H_{220}O_{51}Na$
Mass	1875.9177	2188.1821	2500.4465
Calc. mass	1875.9190	2188.1855	2500.4519
Error (ppm)	0.7	1.6	2.2

(a)

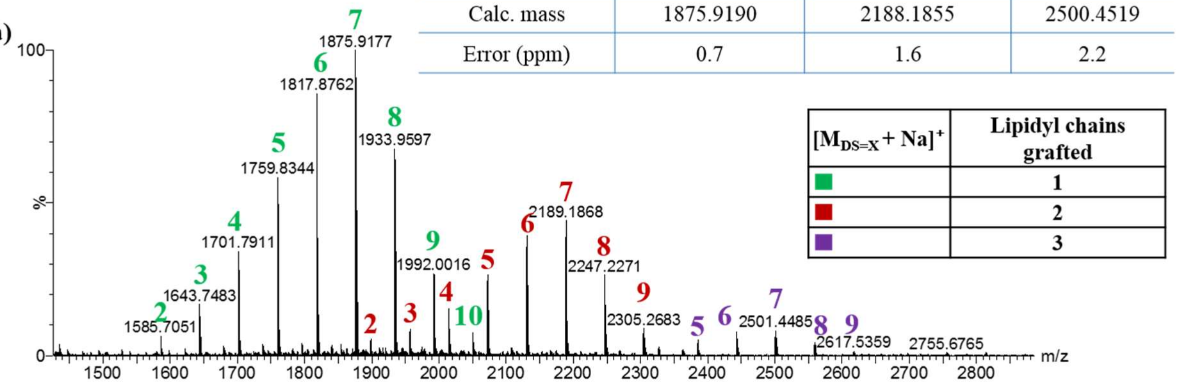

(b)

^{1}H NMR (600 MHz, Pyridine-d_5) δ (ppm): 6.22-5.35 (H_1), 4.80-3.55 (H_2, H_3, H_4, H_5, H_6), 3.62 (-OCH_3), 2.50-1.46 (CH_2 (alkyl chains)), 1.50-1.10 (CH_3 (hydroxypropyl group)), 0.83 (CH_3 (alkyl chains)).

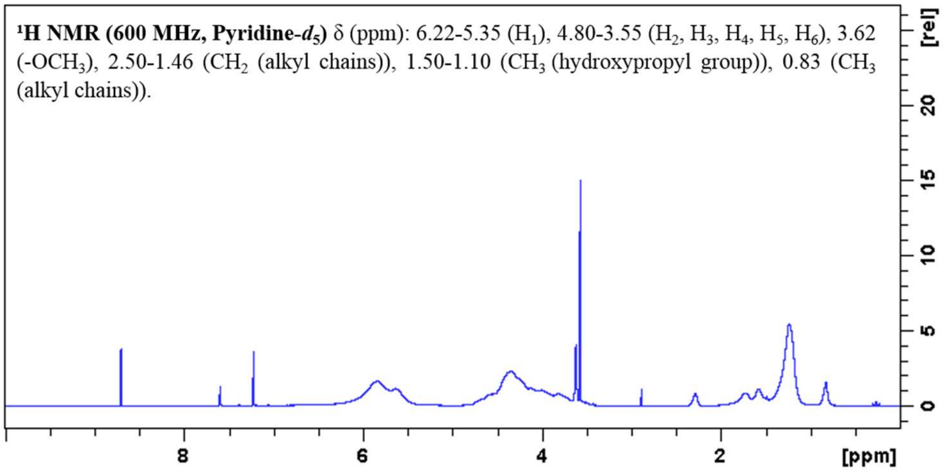

(c)

^{13}C NMR (151 MHz, Pyridine-d_5) δ (ppm): 174.3 (C=O), 105.0-100.0 (C_1), 85.4-78.0 (C_4), 75.7-66.2 (C_2, C_3, C_5), 62.4-61.3 (C_6), 51.6 (-OCH_3), 35.0-23.1 (CH_2 (alkyl chains)), 21.1-19.9 (CH_3 (hydroxypropyl group)), 14.6 (CH_3).

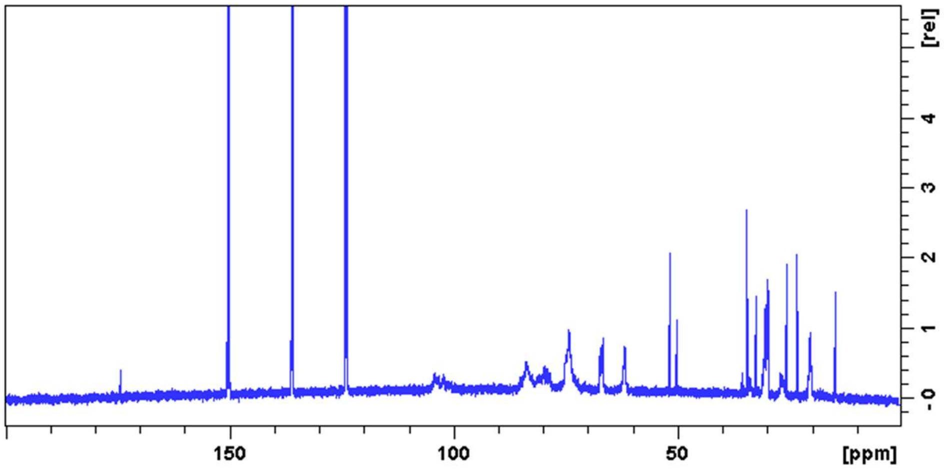

Figure S5: ESI$^+$-HRMS **(a)**, ^{1}H NMR **(b)** and ^{13}C NMR spectra **(c)** of HP-β-CD(C_9)$_2$OOMe **5**.

HP-γ-CD(C9)2OOMe, **6**

$\overline{DS}$: 1.2
MWa: 2031.9 g/mol
Yield: 44%

[M+Na]$^+$	DS$_1$	DS$_2$	DS$_3$
Formula	$C_{85}H_{152}O_{49}Na$	$C_{104}H_{188}O_{52}Na$	$C_{123}H_{224}O_{55}Na$
Mass	1979.9336	2292.1956	2604.4609
Calc. mass	1979.9300	2292.1964	2604.4629
Error (ppm)	1.8	0.3	0.8

(a)

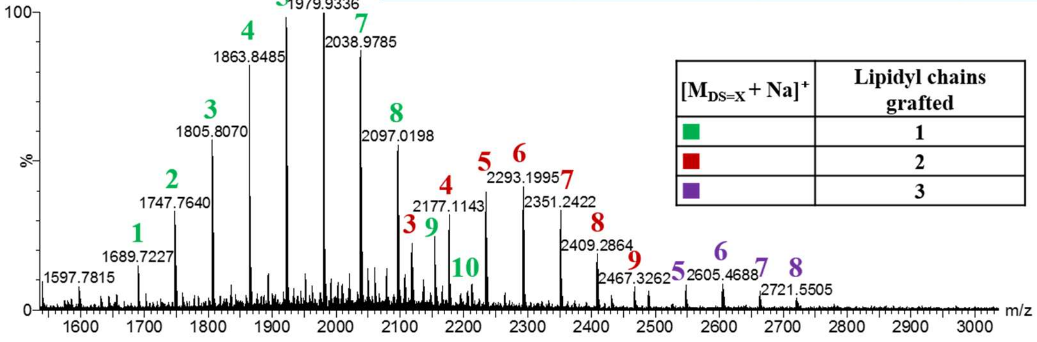

(b) **^{1}H NMR (600 MHz, Pyridine-d_5)** δ (ppm): 6.49-5.31 (H$_1$), 4.70-3.49 (H$_2$, H$_3$, H$_4$, H$_5$, H$_6$), 3.63 (-OCH$_3$), 2.50-1.46 (CH$_2$ (alkyl chains)), 1.44-1.01 (CH$_3$ (hydroxypropyl group)), 0.82 (CH$_3$ (alkyl chains)).

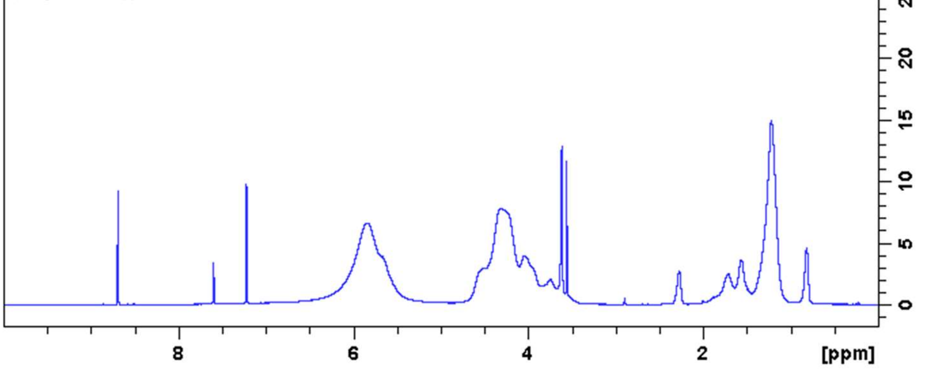

(c) **^{13}C NMR (151 MHz, Pyridine-d_5)** δ (ppm): 174.3 (C=O), 104.6-98.9 (C$_1$), 86.0-78.4 (C$_4$), 75.8-65.6 (C$_2$, C$_3$, C$_5$), 63.0-60.2 (C$_6$), 51.7 (-OCH$_3$), 34.9-22.7 (CH$_2$ (alkyl chains)), 20.9-19.6 (CH$_3$ (hydroxypropyl group)), 14.6 (CH$_3$).

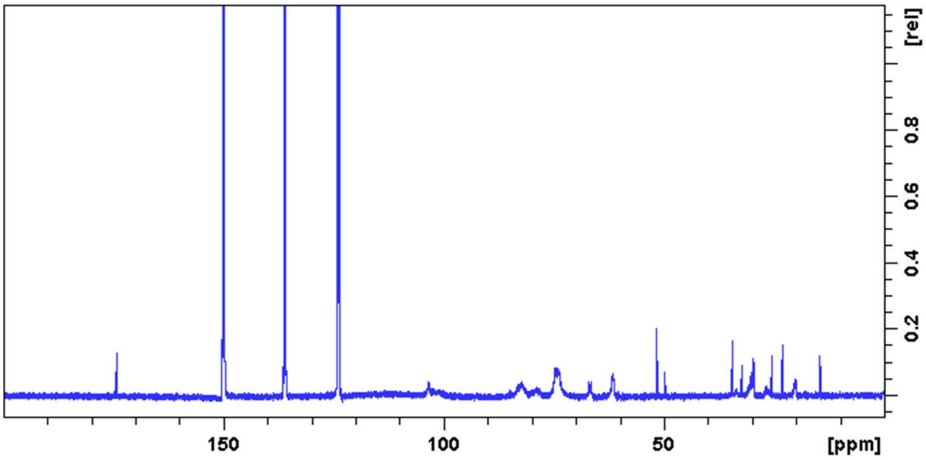

Figure S6: ESI$^+$-HRMS **(a)**, ^{1}H NMR **(b)** and ^{13}C NMR spectra **(c)** of HP-γ-CD(C9)2OOMe **6**.

α-CD(C9)2OOH, **7**

MWa: 1271.0 g/mol
Yield: 78%

[M+Na]+	DS_1
Formula	$C_{54}H_{94}O_{33}Na$
Mass	1293.5549
Calc. mass	1293.5575
Error (ppm)	2.0

(a)

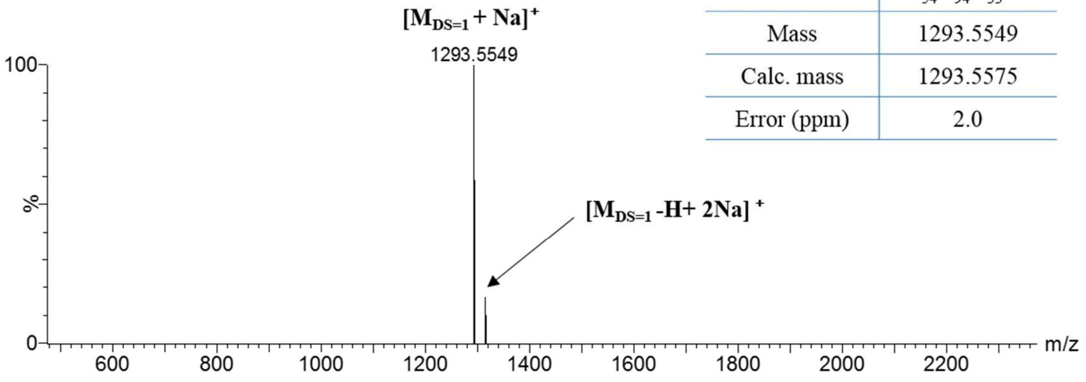

(b)

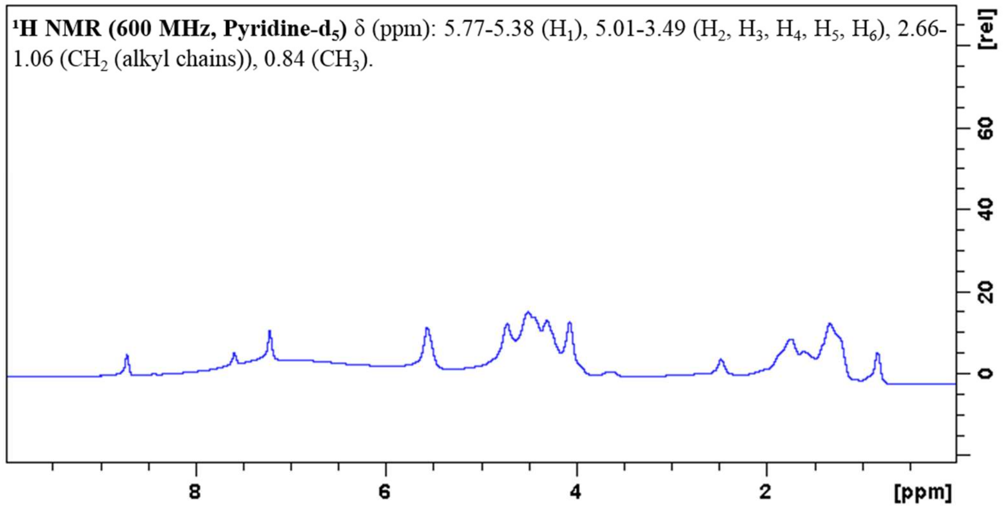

(c) **^{13}C NMR (151 MHz, Pyridine-*d_5*)** δ (ppm): 176.7 (C=O), 104.7-103.1 (C_1), 85.7-81.9 (C_4), 76.3-69.4 (C_2, C_3, C_5), 62.7-60.8 (C_6), 35.9-22.9 (CH_2 (alkyl chains)), 14.6 (CH_3).

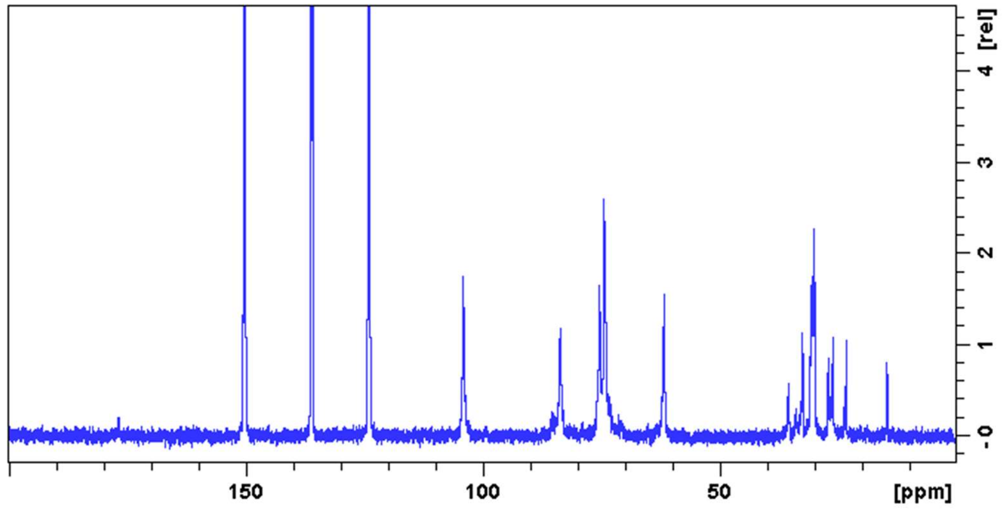

Figure S7: ESI+-HRMS **(a)**, ^{1}H NMR **(b)** and ^{13}C NMR spectra **(c)** of α-CD(C9)2OOH **7**.

β-CD(C9)2OOH, **8**

MWa: 1433.0 g/mol
Yield: 69%

[M+Na]+	DS_1
Formula	$C_{60}H_{104}O_{38}Na$
Mass	1455.6115
Calc. mass	1455.6103
Error (ppm)	0.8

(a)

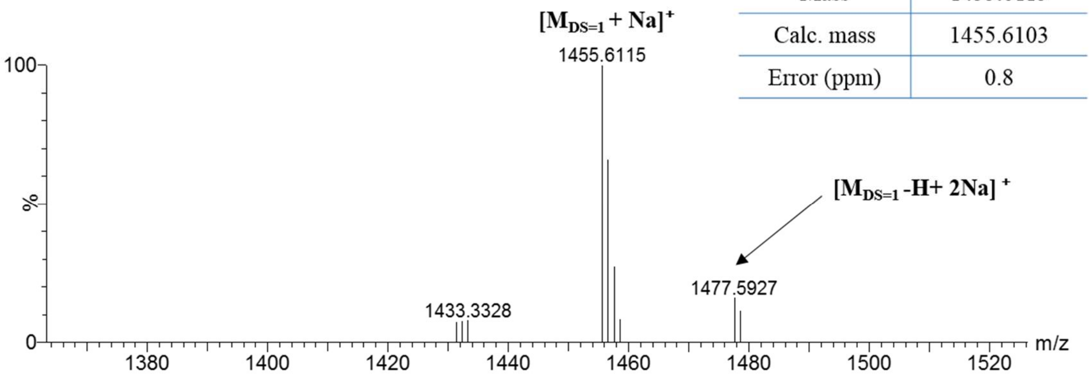

(b)

^{1}H NMR (600 MHz, Pyridine-d_5) δ (ppm): 5.67(H_1), 4.97-3.49 (H_2, H_3, H_4, H_5, H_6), 2.56-1.01 (CH_2 (alkyl chains)), 0.81 (CH_3).

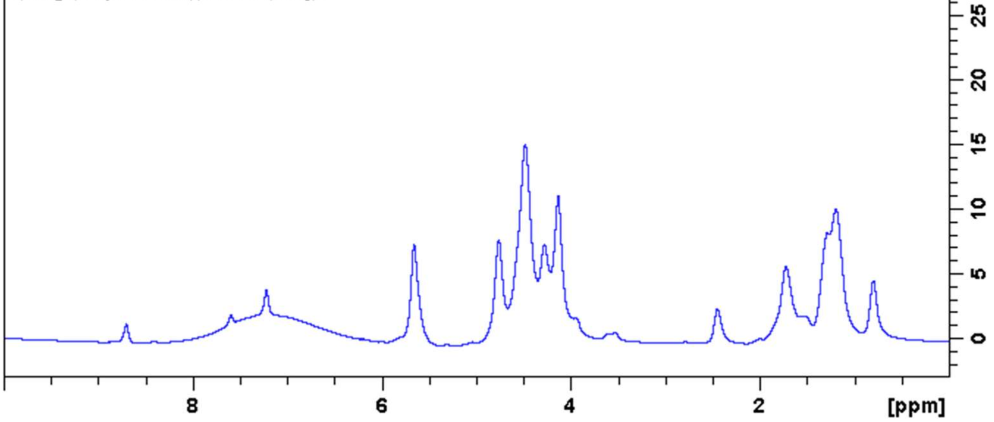

(c)

^{13}C NMR (151 MHz, Pyridine-d_5) δ (ppm): 176.5 (C=O), 104.3 (C_1), 85.4-82.7 (C_4), 75.5-72.2 (C_2, C_3, C_5), 62.5-61.2 (C_6), 35.4-22.9 (CH_2 (alkyl chains)), 14.6 (CH_3).

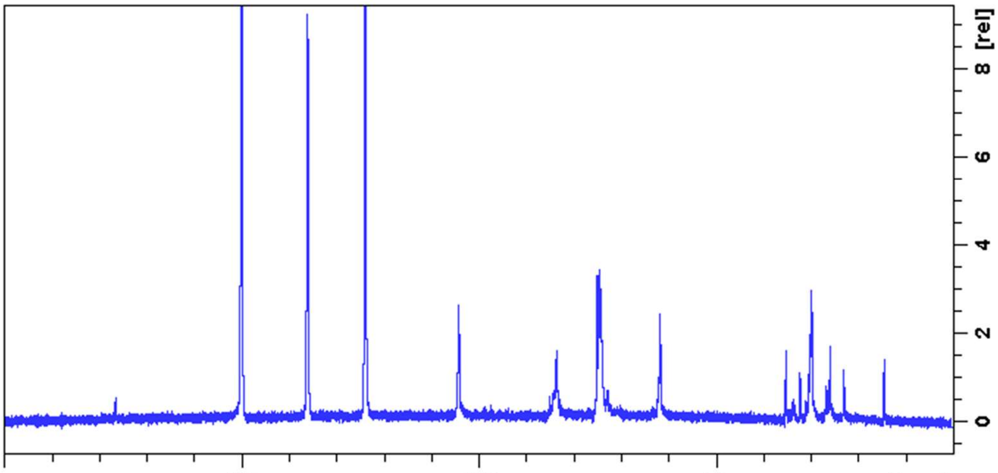

Figure S8: ESI+-HRMS **(a)**, ^{1}H NMR **(b)** and ^{13}C NMR spectra **(c)** of β-CD(C9)2OOH **8**.

γ-CD(C9)2OOH, 9

(a)

MWa: 1595.0 g/mol

Yield: 89%

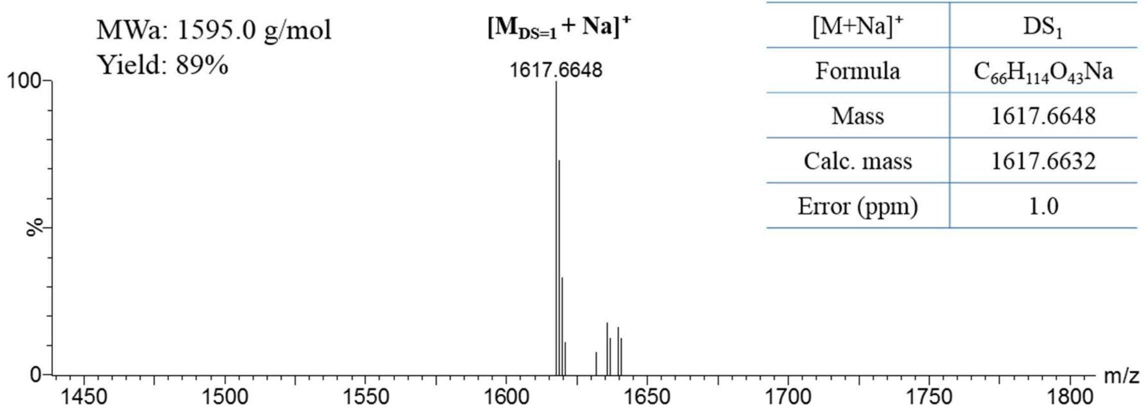

[M+Na]$^+$	DS$_1$
Formula	$C_{66}H_{114}O_{43}Na$
Mass	1617.6648
Calc. mass	1617.6632
Error (ppm)	1.0

(b)

^{1}H NMR (600 MHz, Pyridine-*d*$_5$) δ (ppm): 6.03-5.20 (H_1), 5.10-3.47 (H_2, H_3, H_4, H_5, H_6), 2.67-1.00 (CH_2 (alkyl chains)), 0.84 (CH_3).

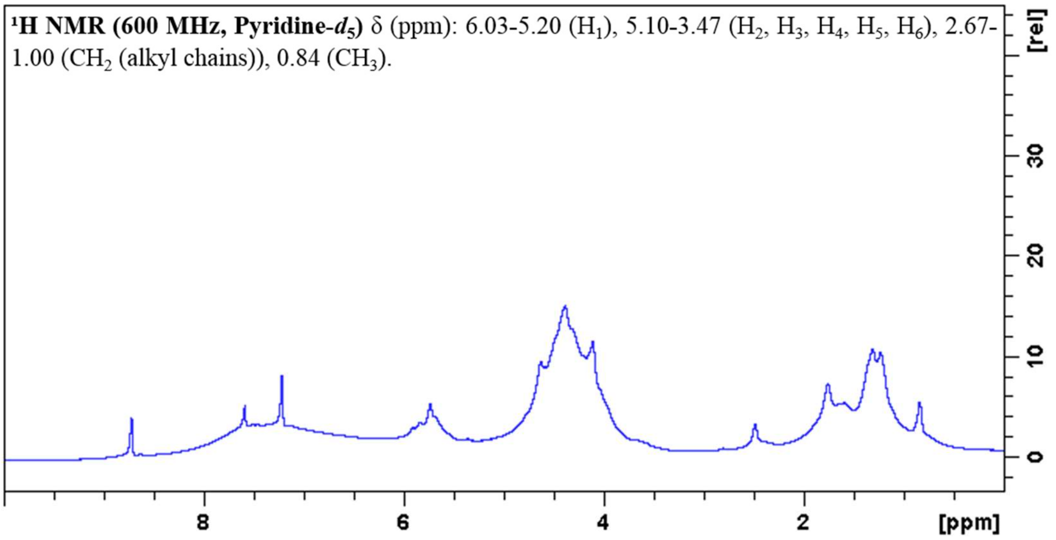

(c)

^{13}C NMR (151 MHz, Pyridine-*d*$_5$) δ (ppm): 176.6 (C=O), 105.2-94.6 (C_1), 85.2-78.2 (C_4), 76.7-68.6 (C_2, C_3, C_5), 64.9-60.2 (C_6), 36.0-22.4 (CH_2 (alkyl chains)), 14.6 (CH_3).

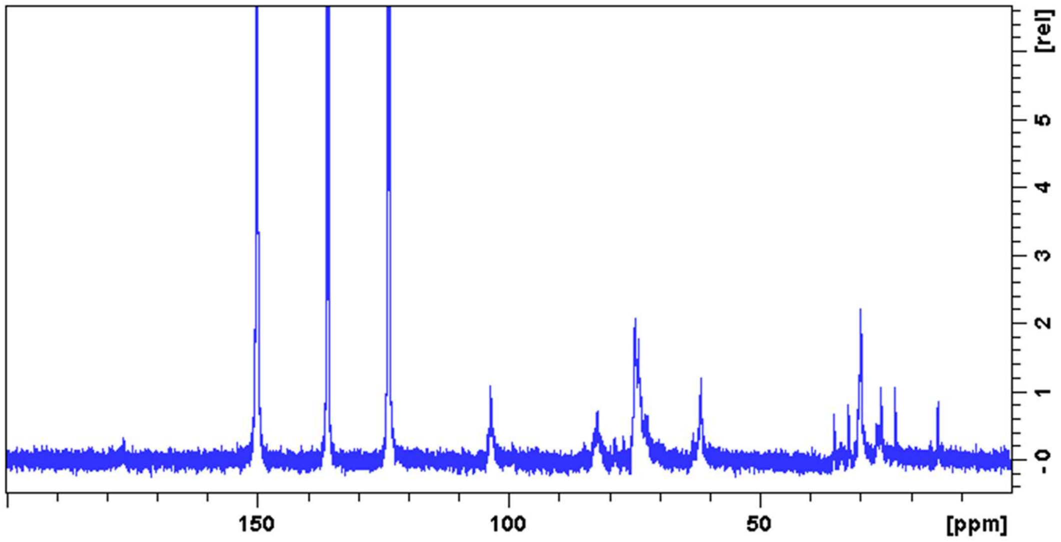

Figure S9: ESI$^+$-HRMS **(a)**, ^{1}H NMR **(b)** and ^{13}C NMR spectra **(c)** of γ-CD(C9)2OOH **9**.

HP-α-CD$(C_9)_2$OOH**, 10**

$\overline{DS}$ (hydroxypropyl group): 3.9
MWa: 1499.5 g/mol
Yield: 82%

$[M+Na]^+$	DS_1
Formula	$C_{66}H_{118}O_{37}Na$
Mass	1525.7257
Calc. mass	1525.7250
Error (ppm)	0.5

$[M_{DS=X} + Na]^+$	Lipidyl chains grafted
■	1

(a)

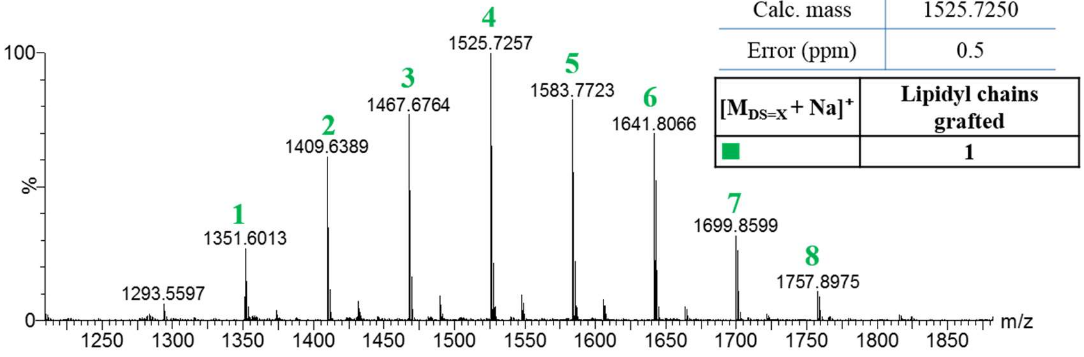

(b) **^{1}H NMR (600 MHz, Pyridine-d_5)** δ (ppm): 5.72-5.31 (H_1), 4.89-3.31 (H_2, H_3, H_4, H_5, H_6), 2.78-1.47 (CH_2 (alkyl chains)), 1.50-1.00 (CH_3 (hydroxypropyl group)), 0.84 (CH_3 (alkyl chains)).

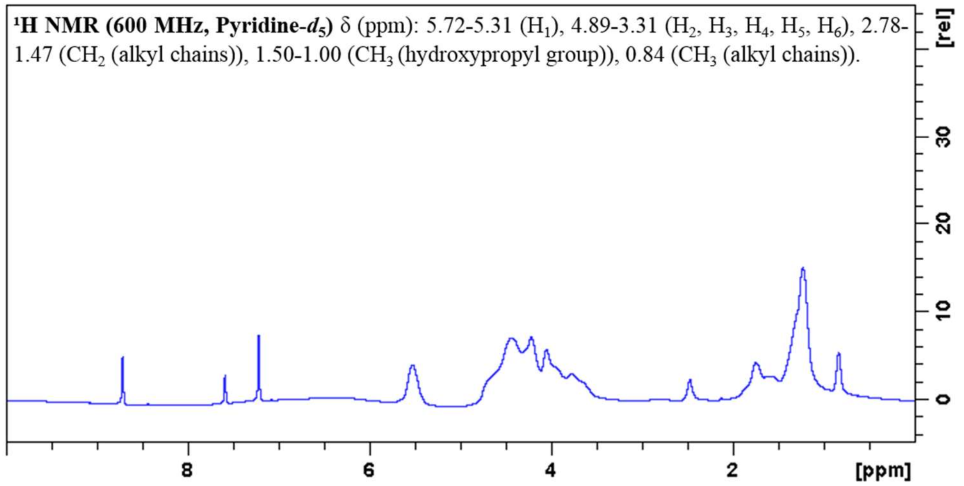

(c) **^{13}C NMR (151 MHz, Pyridine-d_5)** δ (ppm): 176.7 (C=O), 105.2-100.0 (C_1), 87.3-76.8 (C_4), 76.6-65.1 (C_2, C_3, C_5), 63.8-60.4 (C_6), 36.6-22.4 (CH_2 (alkyl chains)), 21.5-18.8 (CH_3 (hydroxypropyl group)), 14.6 (CH_3).

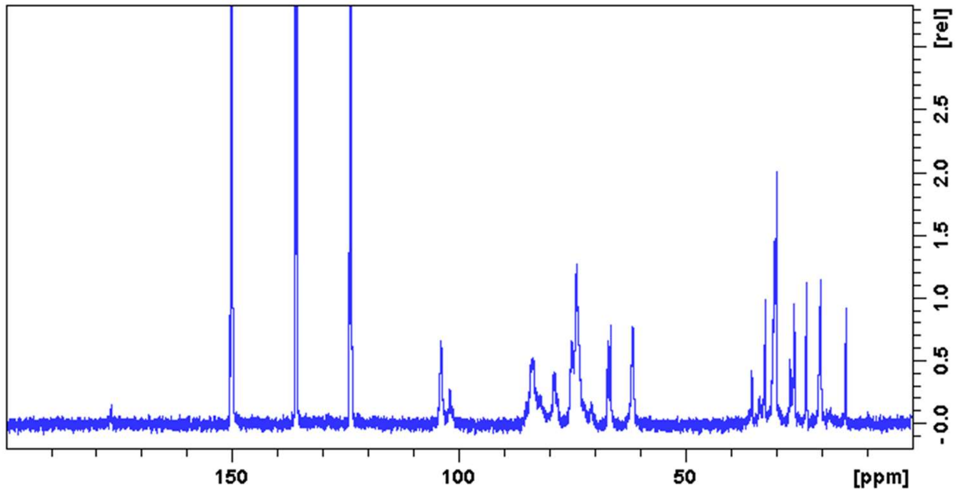

Figure S10: ESI$^+$-HRMS **(a)**, ^{1}H NMR **(b)** and ^{13}C NMR spectra **(c)** of HP-α-CD$(C_9)_2$OOH **10**.

HP-β-CD(C9)2OOH, **11**

$\overline{DS}$ (hydroxypropyl group): 5.2
MWa: 1736.3 g/mol
Yield: 84%

$[M+Na]^+$	DS_1
Formula	$C_{81}H_{146}O_{45}Na$
Mass	1861.9044
Calc. mass	1861.9034
Error (ppm)	0.5

$[M_{DS=X} + Na]^+$	**Lipidyl chains grafted**
■	**1**

(a)

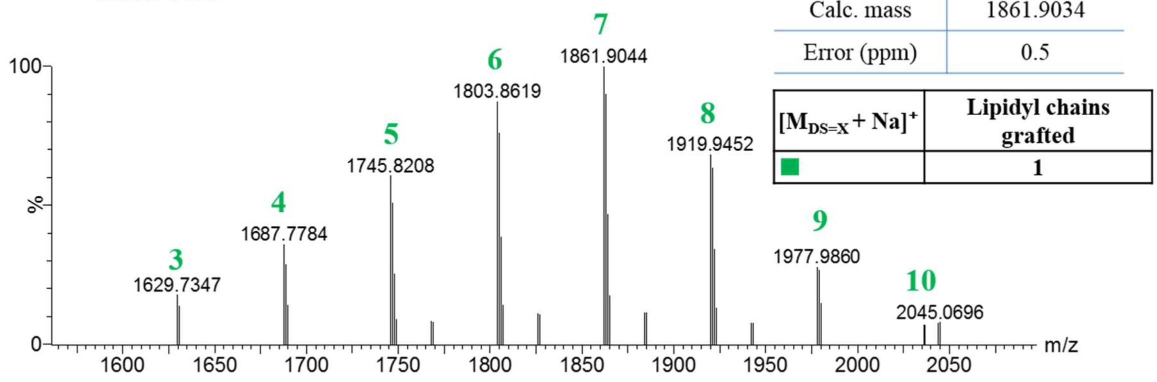

(b) **^{1}H NMR (600 MHz, Pyridine-d_5)** δ (ppm): 6.00-5.35 (H_1), 5.09-3.09 (H_2, H_3, H_4, H_5, H_6), 2.96-1.57 (CH_2 (alkyl chains)), 1.56-1.00 (CH_3 (hydroxypropyl group)), 0.84 (CH_3 (alkyl chains)).

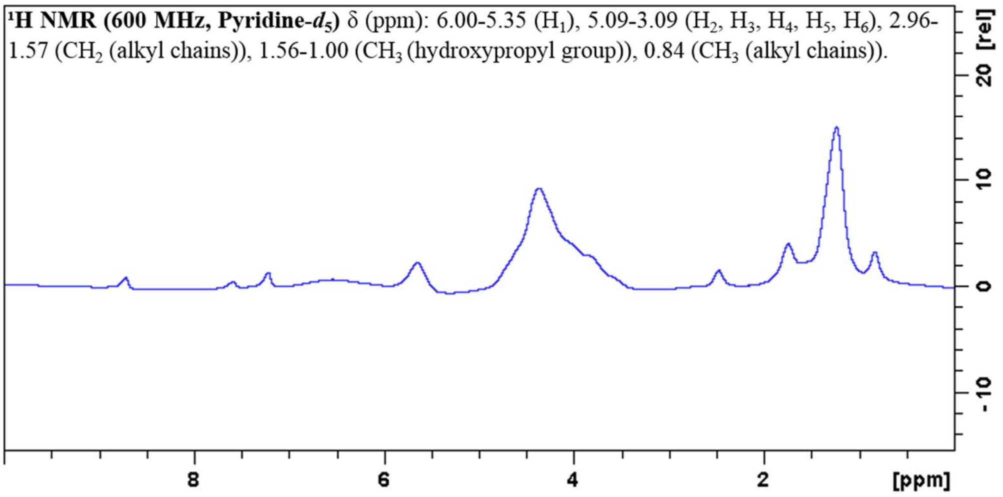

(c) **^{13}C NMR (151 MHz, Pyridine-d_5)** δ (ppm): 176.6 (C=O), 105.2-100.0 (C_1), 85.6-77.5 (C_4), 75.7-65.7 (C_2, C_3, C_5), 63.1-60.2 (C_6), 36.2-22.6 (CH_2 (alkyl chains)), 21.3-19.8 (CH_3 (hydroxypropyl group)), 14.6 (CH_3).

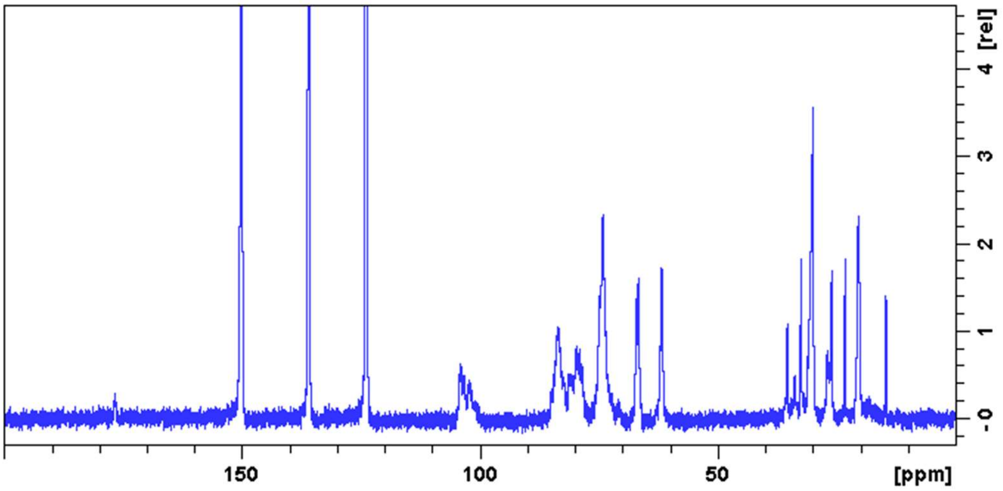

Figure S11: ESI$^+$-HRMS **(a)**, ^{1}H NMR **(b)** and ^{13}C NMR spectra **(c)** of HP-β-CD(C9)2OOH **11**.

HP-γ-CD(C_9)$_2$OOH, **12**

[M+Na]$^+$	DS_1
Formula	$C_{84}H_{150}O_{49}Na$
Mass	1965.9083
Calc. mass	1965.9143
Error (ppm)	3.1

$\overline{DS}$ (hydroxypropyl group): 5.0
MWa: 1887.3 g/mol
Yield: 89%

[$M_{DS=x}$ + Na]$^+$	Lipidyl chains grafted
■	1

(a)

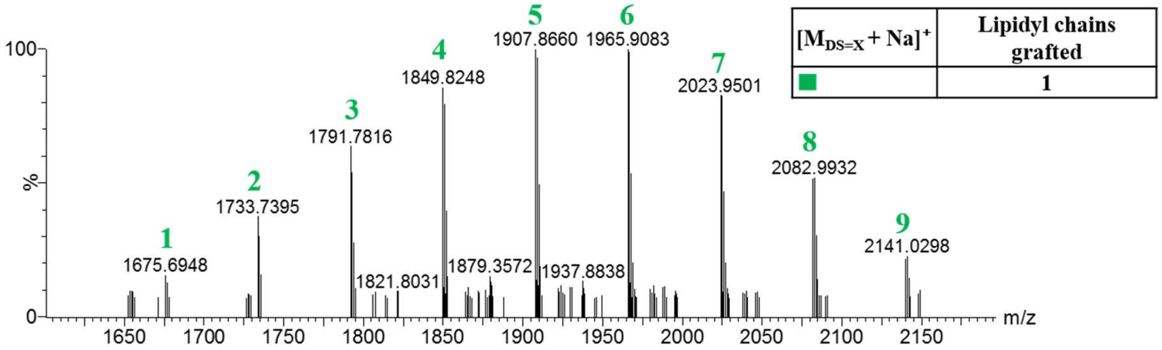

(b)

^{1}H NMR (600 MHz, Pyridine-d_5) δ (ppm): 5.99-5.27 (H_1), 4.80-3.24 (H_2, H_3, H_4, H_5, H_6), 2.60-1.47 (CH_2 (alkyl chains)), 1.47-1.02 (CH_3 (hydroxypropyl group)), 0.83 (CH_3 (alkyl chains)).

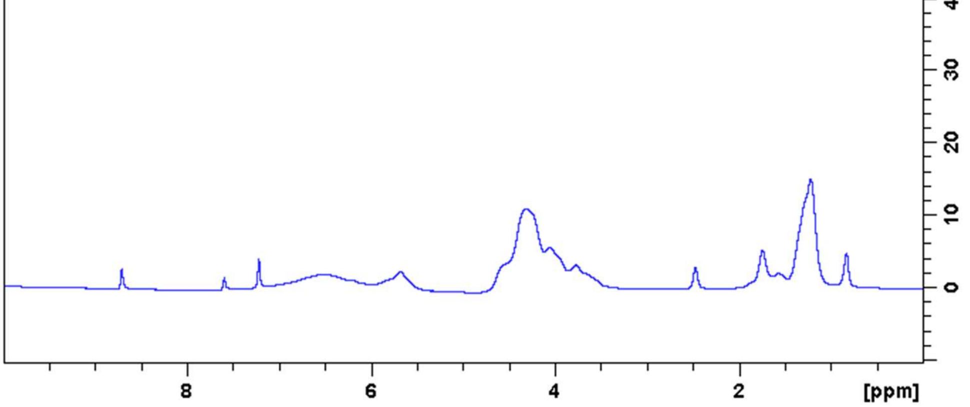

(c)

^{13}C NMR (151 MHz, Pyridine-d_5) δ (ppm): 176.5 (C=O), 104.4-98.8 (C_1), 85.9-77.6 (C_4), 76.2-65.7 (C_2, C_3, C_5), 62.9-60.4 (C_6), 35.7-22.8 (CH_2 (alkyl chains)), 20.9-19.6 (CH_3 (hydroxypropyl group)), 14.6 (CH_3).

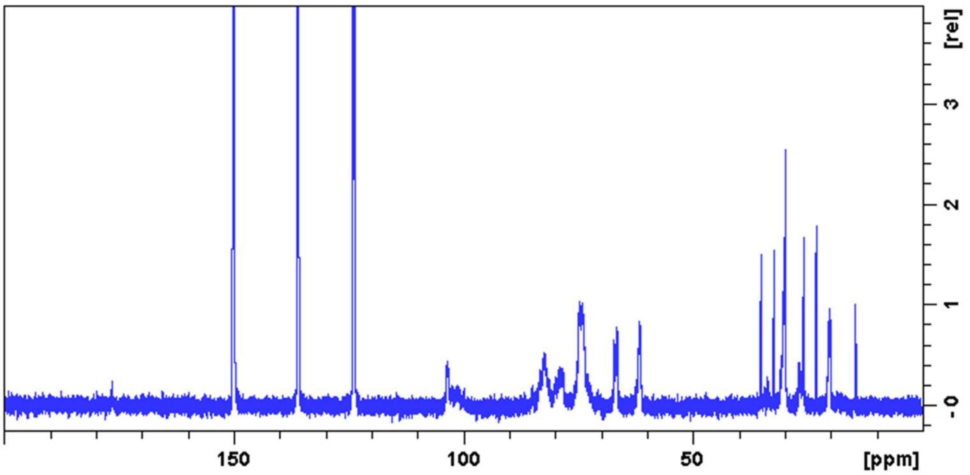

Figure S12: ESI$^+$-HRMS **(a)**, ^{1}H NMR **(b)** and ^{13}C NMR spectra **(c)** of HP-γ-CD(C_9)$_2$OOH **12**.

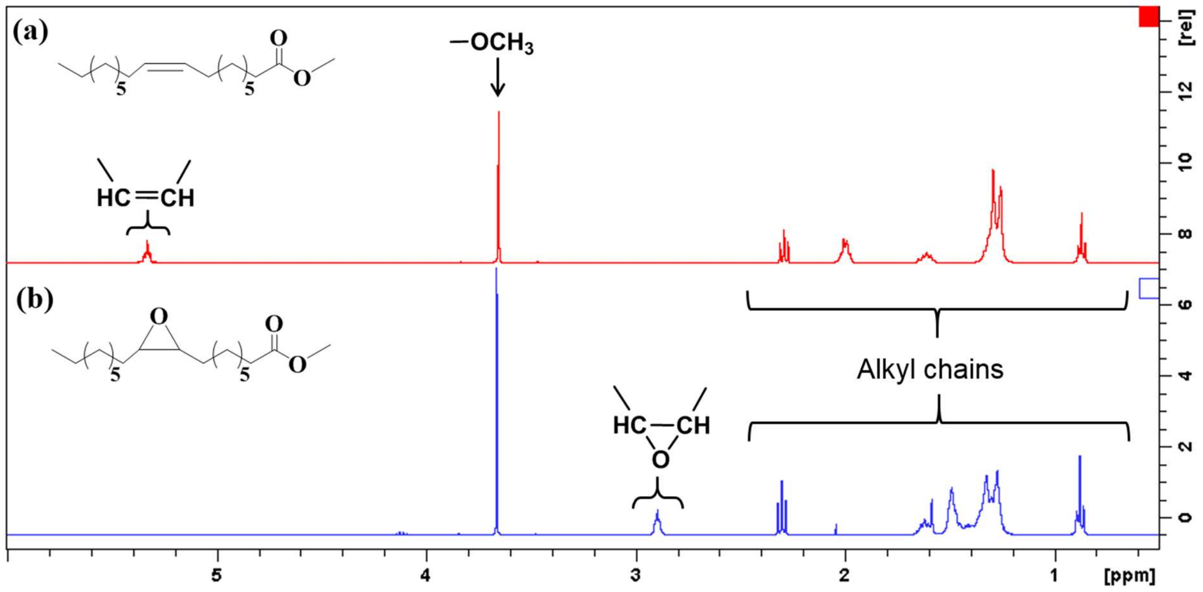

Figure S13: ^{1}H NMR spectra of methyl oleate **(a)** and of epoxidated methyl oleate **(b)** (400 MHz, $CDCl_3$, 298 K).

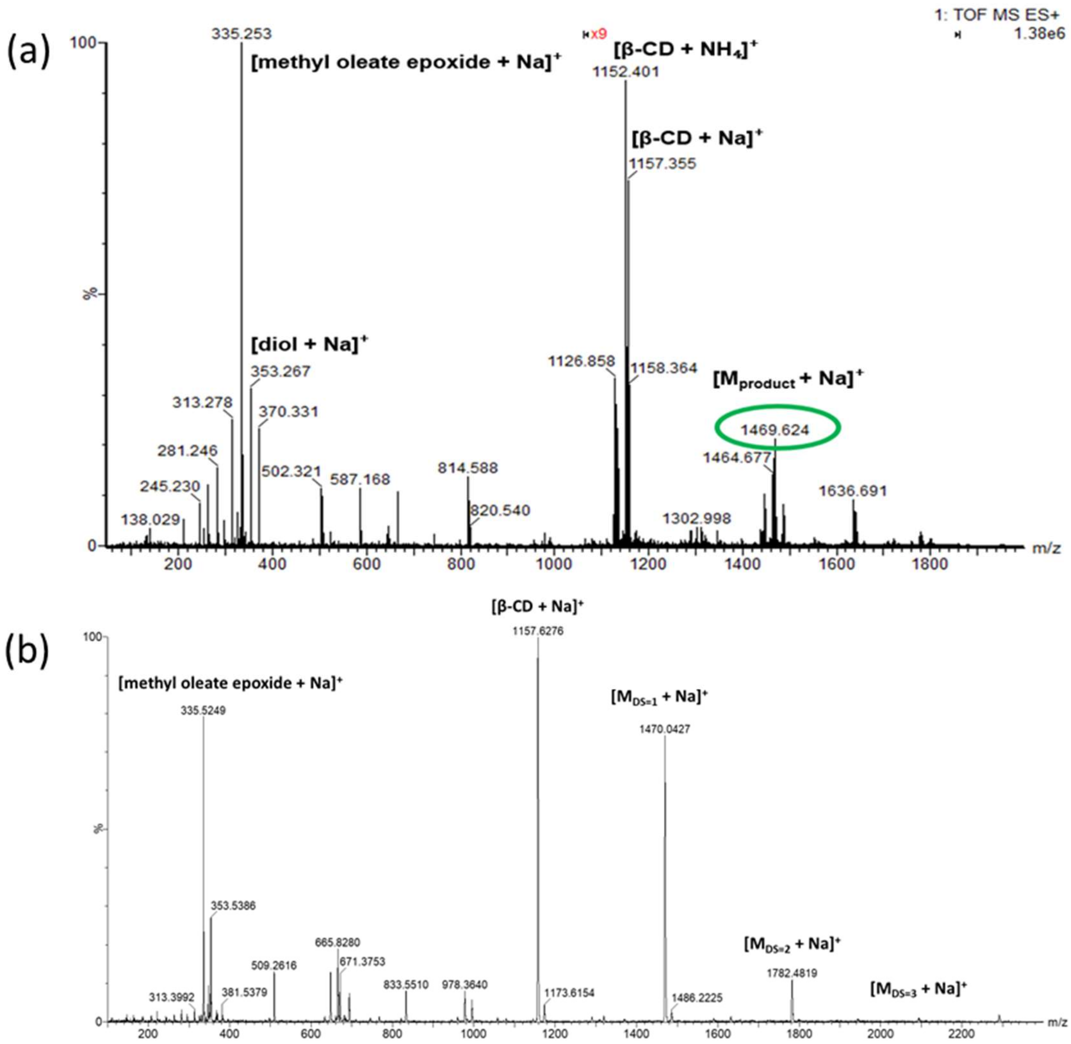

Figure S14: ESI^{+}-MS spectra of the ball milling reaction medium of the methyl oleate epoxide opening with the β-CD assisted by APTS (a) and H_2SO_4 (b). The spectra highlight the presence of the expected grafted species and also the starting materials (epoxide, β-CD).

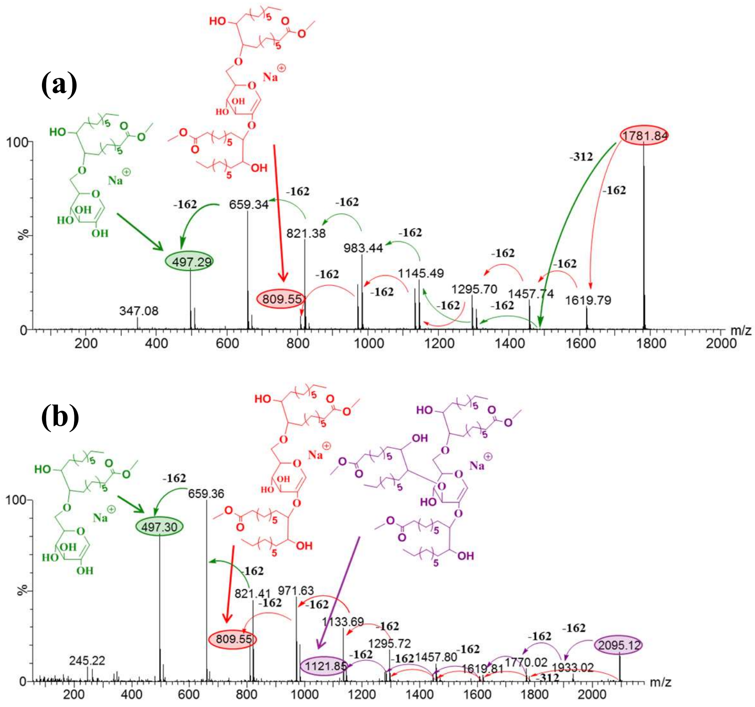

Figure S15: ESI⁺-MS/MS spectra of the $[M+Na]^+$ ions of DS=2 **(a)** and DS=3 **(b)** species of β-CD$(C_9)_2$OOMe **1** obtained from ball milling.

$[M_{DS=X} + Na]^+$ (X = number of HP substituents)	Lipidyl chains grafted
■	1
■	2
■	3

$$DS = \frac{1 \times \sum I_1 + 2 \times \sum I_2 + 3 \times \sum I_3}{\sum_i I_i} = 1.3$$

$$MW_a = MW_{a_{HP-\beta-CD}} + DS \times MW_{epoxide}$$

$$MW_a = 1490.5 + 1{,}3 \times 312.5 = 1892.5\ g/mol$$

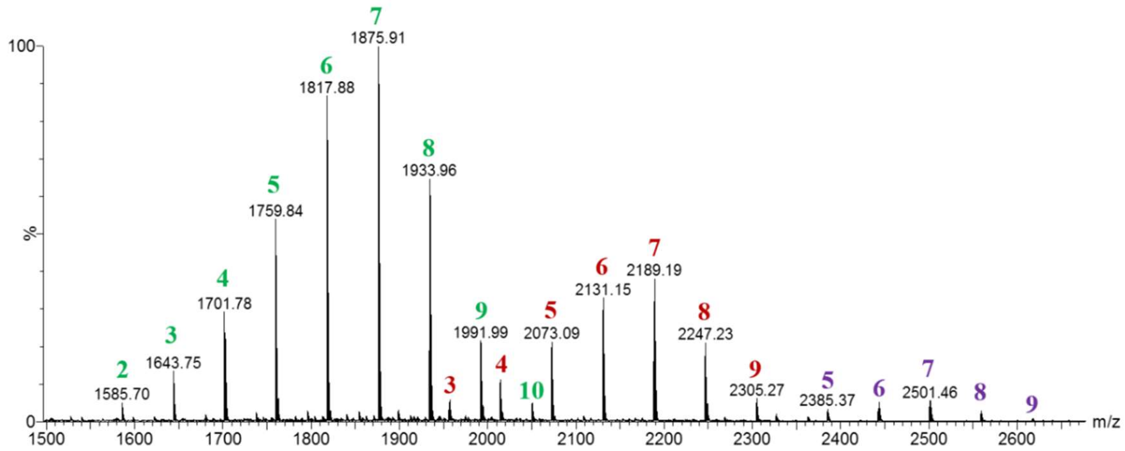

Figure S16: ESI⁺-MS spectrum of the of HPβ-CD$(C_9)_2$OOMe **5** obtained from ball milling, showing mono (green), di (red) and tri (blue) grafted species.

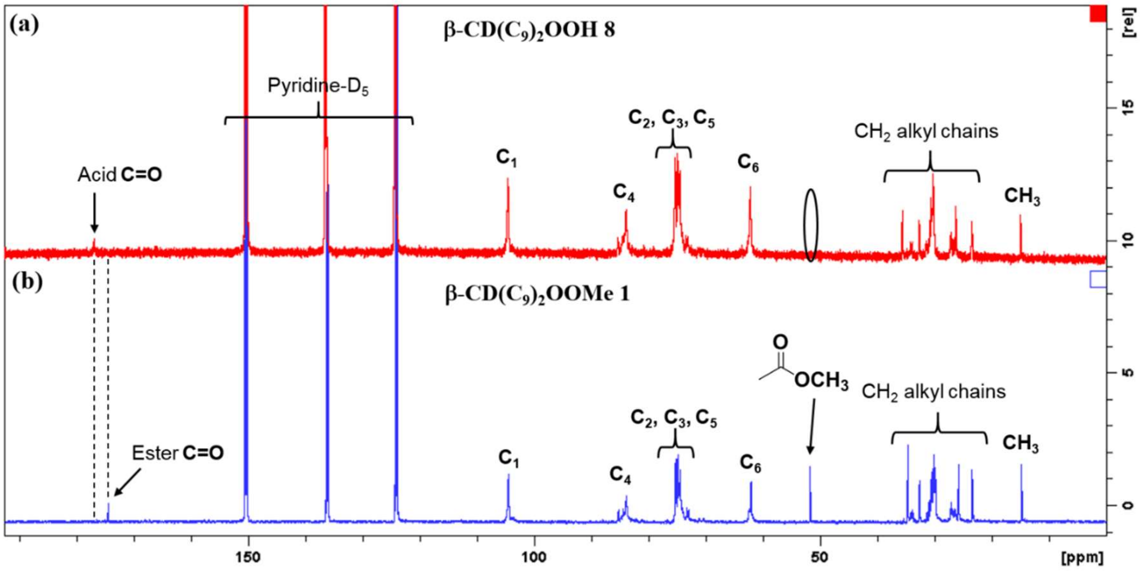

Figure S17: ^{13}C NMR spectra of β-CD$(C_9)_2$OOH **8 (a)** and of β-CD$(C_9)_2$OOMe **1 (b)** (151 MHz, pyridine-D_5, 298 K).

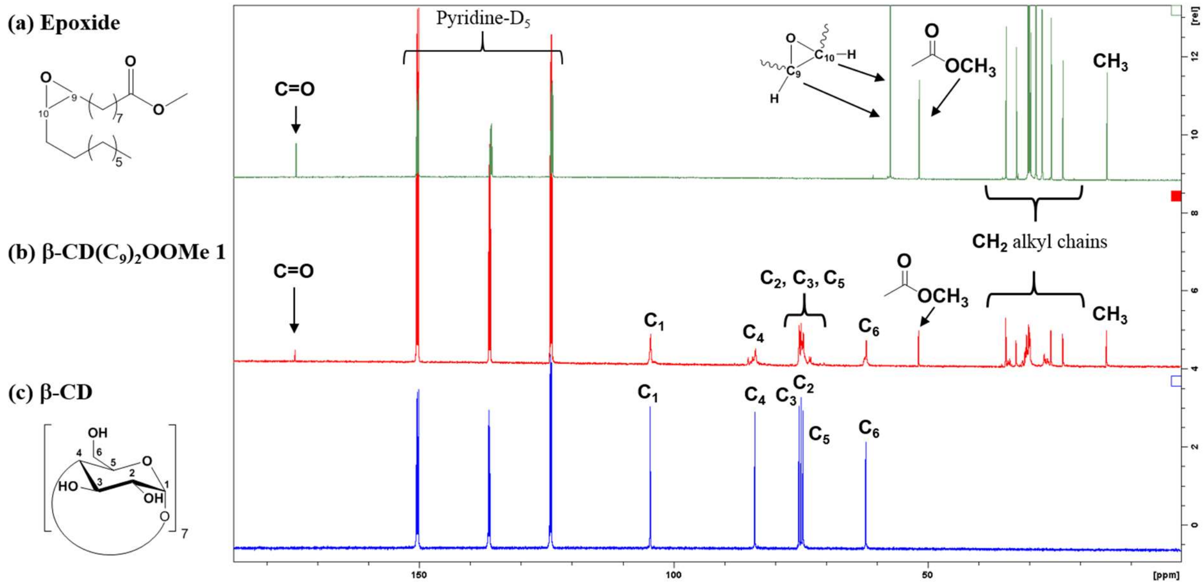

Figure S18: ^{13}C NMR spectra of methyl oleate epoxide **(a)**, β-CD(C_9)$_2$OOMe **1 (b)** and β-CD native **(c)** (151 MHz, pyridine-D_5, 298 K).

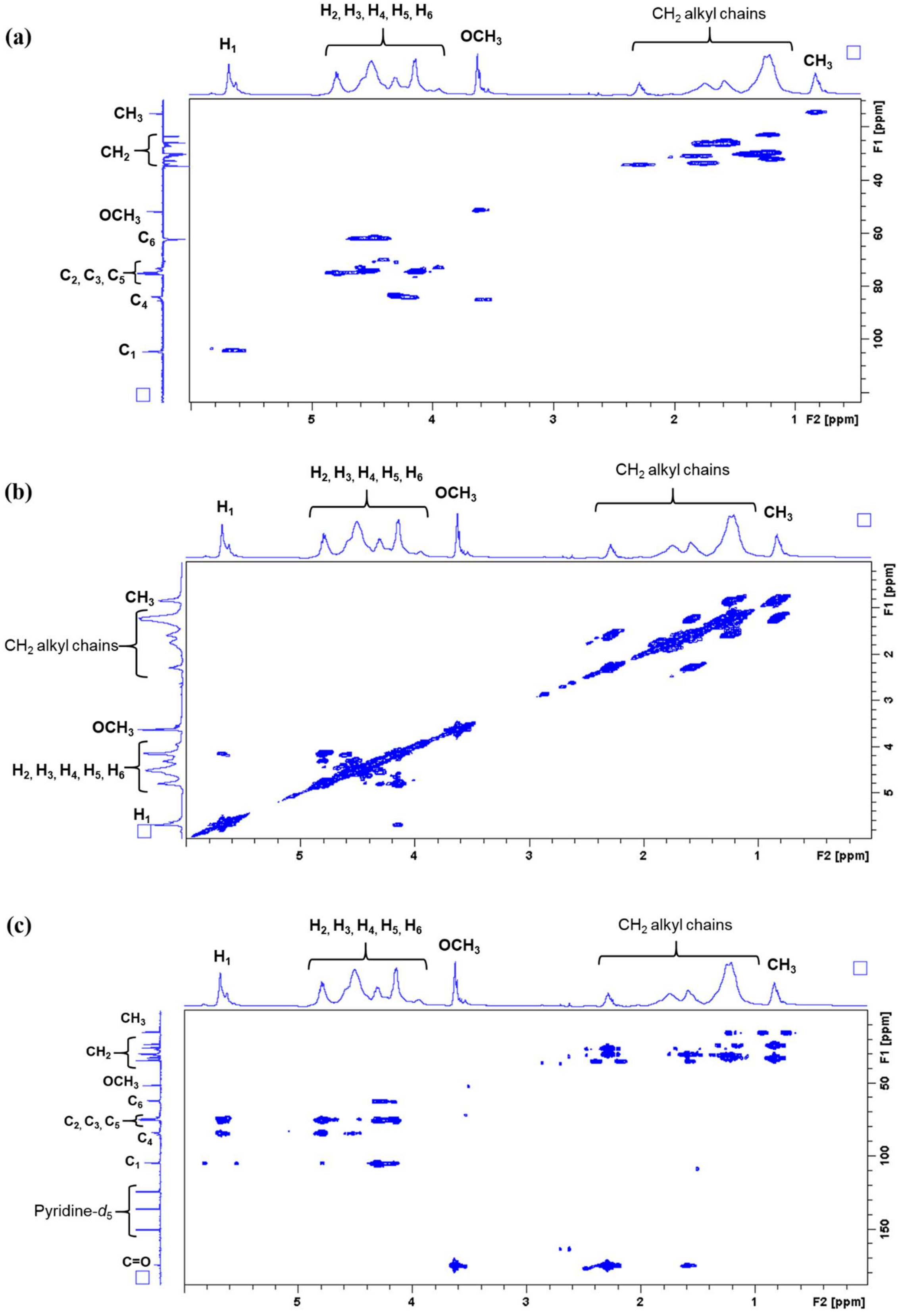

Figure S19:2D NMR spectra of **1**: HSQC **(a)**, COSY **(b)** and HMBC **(c)** (600 MHz, pyridine-D_5, 298 K).

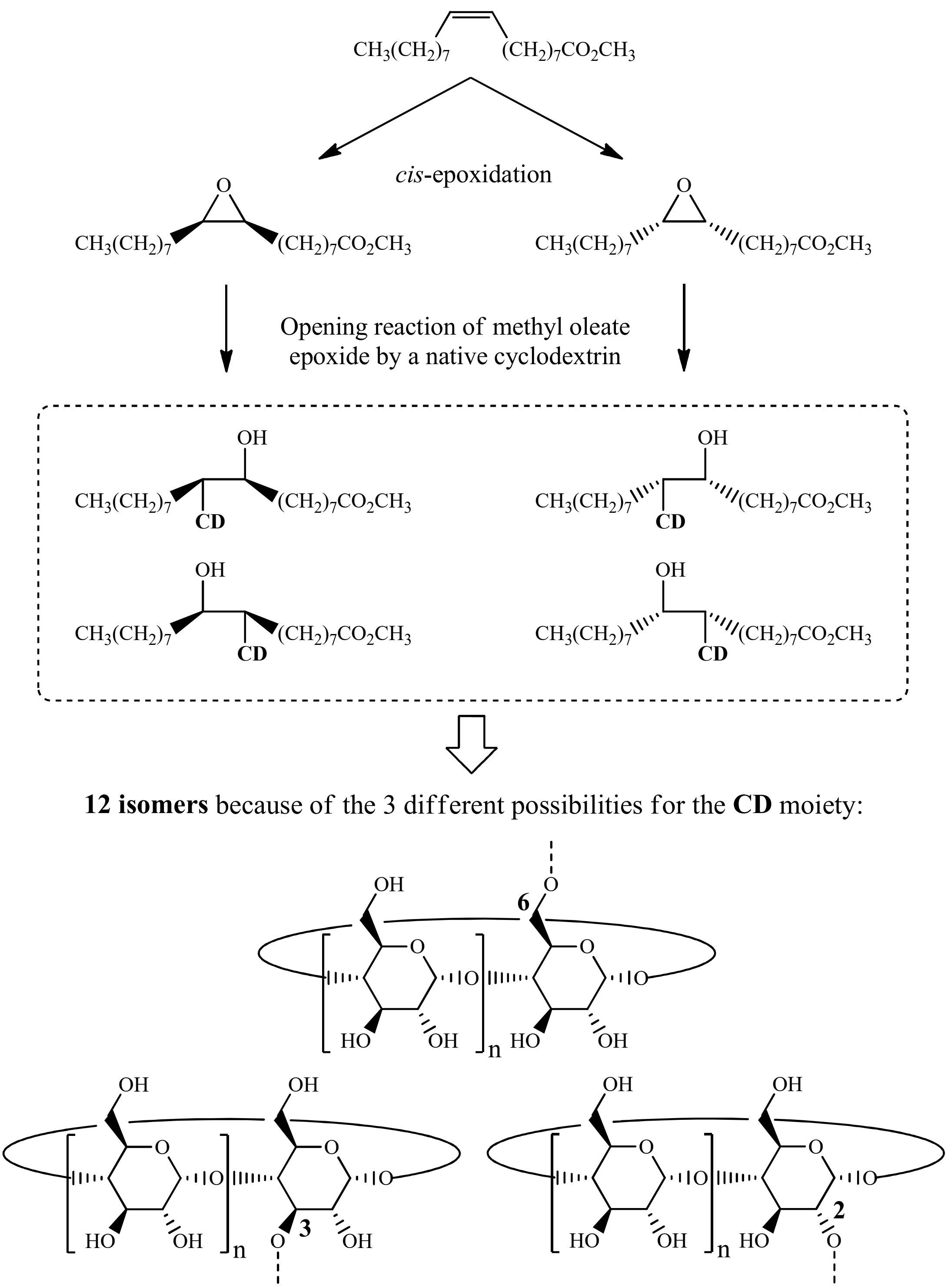

Figure S20: Opening reaction of epoxide by free cyclodextrin.

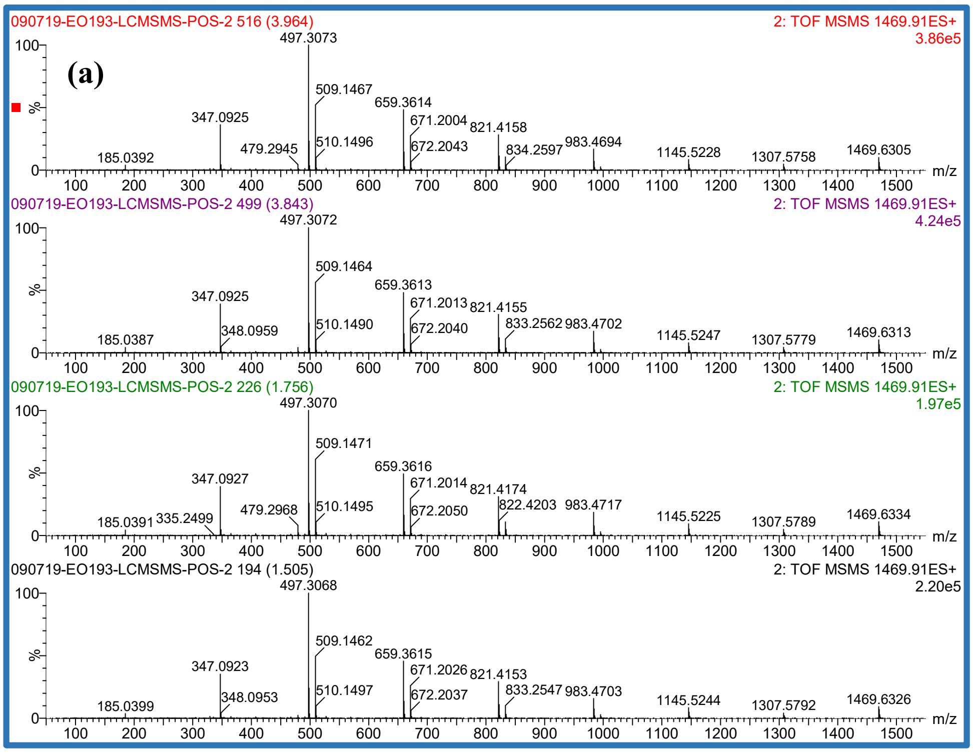
(a)
090719-EO193-LCMSMS-POS-2 516 (3.964)
2: TOF MSMS 1469.91ES+
3.86e5
497.3073
509.1467
659.3614
347.0925
671.2004
821.4158
479.2945
510.1496
672.2043
834.2597
983.4694
185.0392
1145.5228
1307.5758
1469.6305
090719-EO193-LCMSMS-POS-2 499 (3.843)
2: TOF MSMS 1469.91ES+
4.24e5
497.3072
509.1464
659.3613
347.0925
671.2013
821.4155
348.0959
510.1490
672.2040
833.2562
983.4702
185.0387
1145.5247
1307.5779
1469.6313
090719-EO193-LCMSMS-POS-2 226 (1.756)
2: TOF MSMS 1469.91ES+
1.97e5
497.3070
509.1471
659.3616
347.0927
671.2014
821.4174
479.2968
510.1495
672.2050
822.4203
983.4717
185.0391
335.2499
1145.5225
1307.5789
1469.6334
090719-EO193-LCMSMS-POS-2 194 (1.505)
2: TOF MSMS 1469.91ES+
2.20e5
497.3068
509.1462
659.3615
347.0923
671.2026
821.4153
510.1497
348.0953
672.2037
833.2547
983.4703
185.0399
1145.5244
1307.5792
1469.6326
100
%
0
m/z

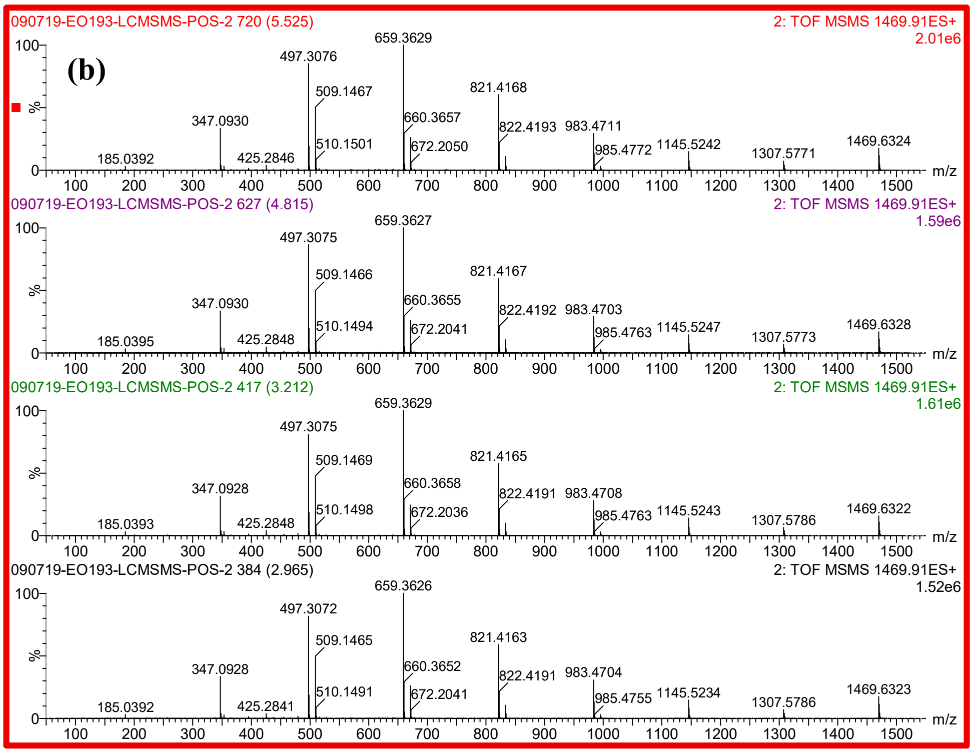
(b)
090719-EO193-LCMSMS-POS-2 720 (5.525)
2: TOF MSMS 1469.91ES+
2.01e6
659.3629
497.3076
509.1467
821.4168
347.0930
660.3657
822.4193
983.4711
510.1501
672.2050
985.4772
1145.5242
185.0392
425.2846
1307.5771
1469.6324
090719-EO193-LCMSMS-POS-2 627 (4.815)
2: TOF MSMS 1469.91ES+
1.59e6
659.3627
497.3075
509.1466
821.4167
347.0930
660.3655
822.4192
983.4703
510.1494
672.2041
985.4763
1145.5247
185.0395
425.2848
1307.5773
1469.6328
090719-EO193-LCMSMS-POS-2 417 (3.212)
2: TOF MSMS 1469.91ES+
1.61e6
659.3629
497.3075
509.1469
821.4165
347.0928
660.3658
822.4191
983.4708
510.1498
672.2036
985.4763
1145.5243
185.0393
425.2848
1307.5786
1469.6322
090719-EO193-LCMSMS-POS-2 384 (2.965)
2: TOF MSMS 1469.91ES+
1.52e6
659.3626
497.3072
509.1465
821.4163
347.0928
660.3652
822.4191
983.4704
510.1491
672.2041
985.4755
1145.5234
185.0392
425.2841
1307.5786
1469.6323
100
%
0
m/z

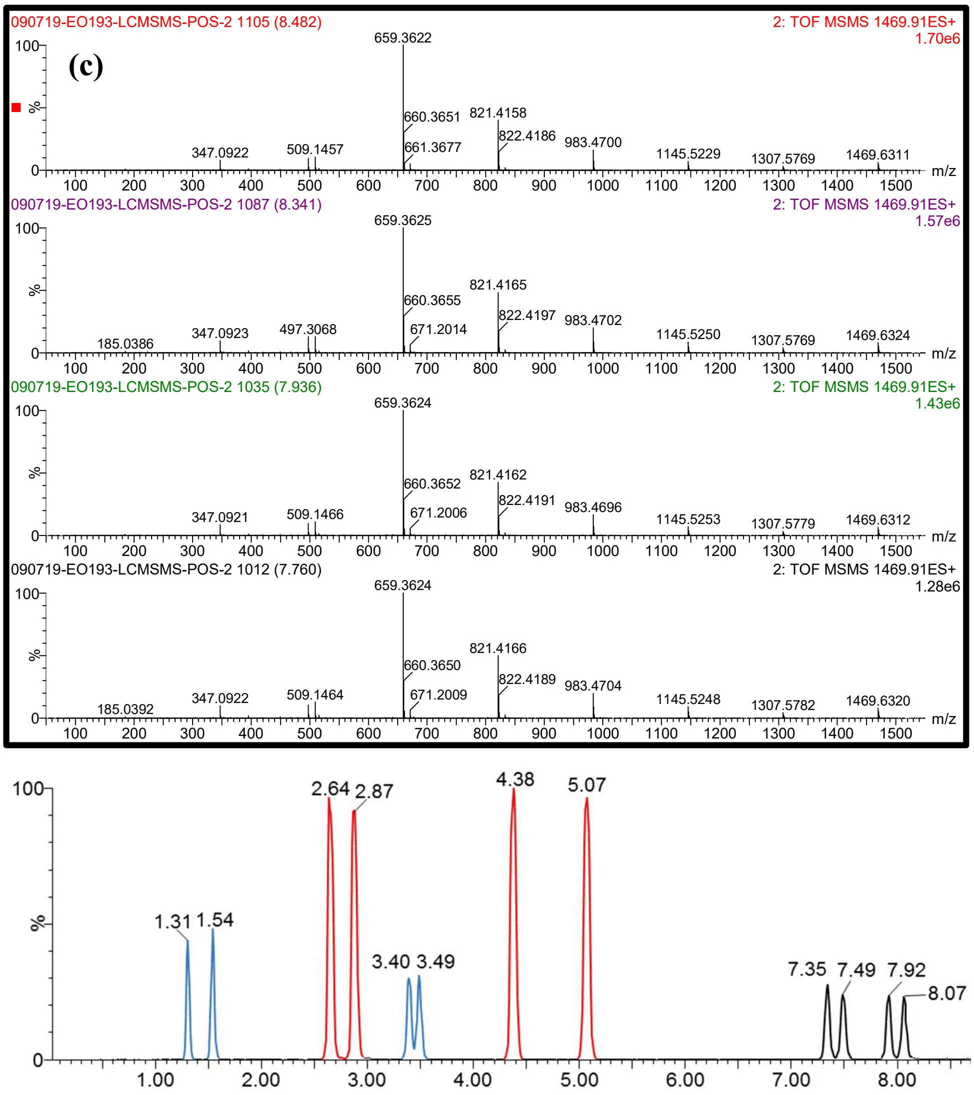

Figure S21: ESI$^+$-MS/MS (95 eV) spectra of the twelve DS=1 isomers ([M+Na]$^+$ m/z 1469.63) of **1** allowing the distinction of 3 groups of regioisomers. **(a)** Blue (R_t 3.49, 3.40, 1.54 and 1.31 min), **(b)** Red (R_t 5.07, 4.38, 2.87 and 2.64 min) and **(c)** Black (R_t 8.07, 7.92, 7.49 and 7.35 min).

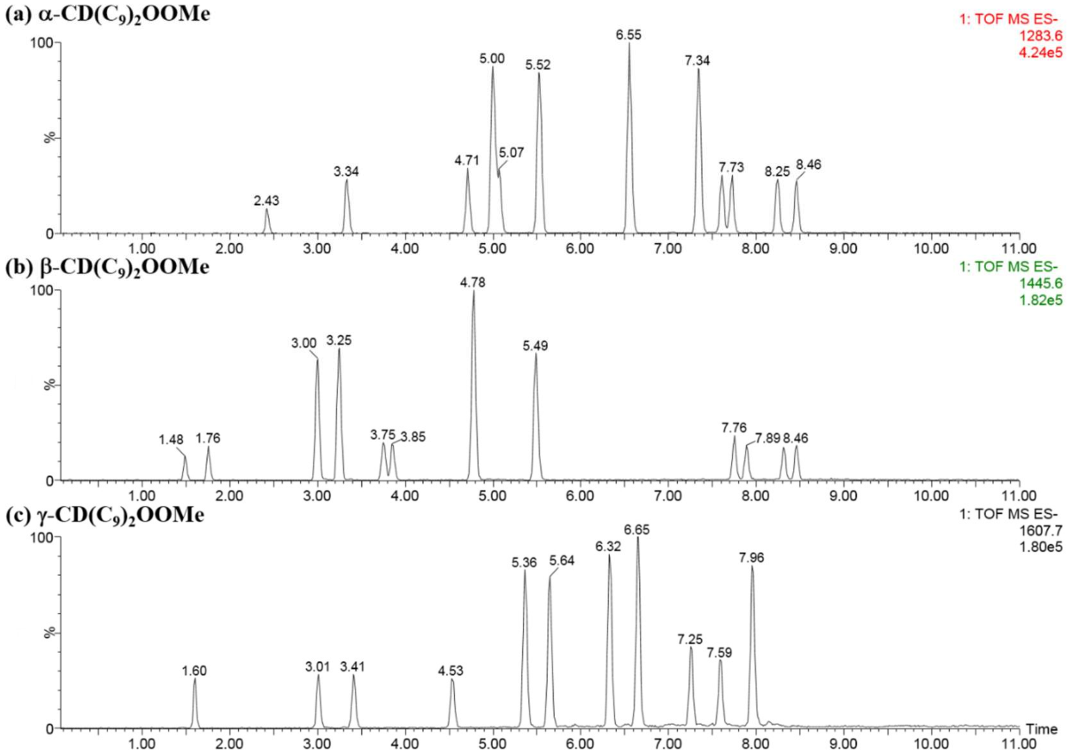

Figure S22: [M-H]- reconstituted ion chromatograms of **2** : DS=1 m/z 1283.58 **(a)** **1** DS=1 m/z 1145.63 **(b)** and **3** DS=1 m/z 1607.68 **(c)**.

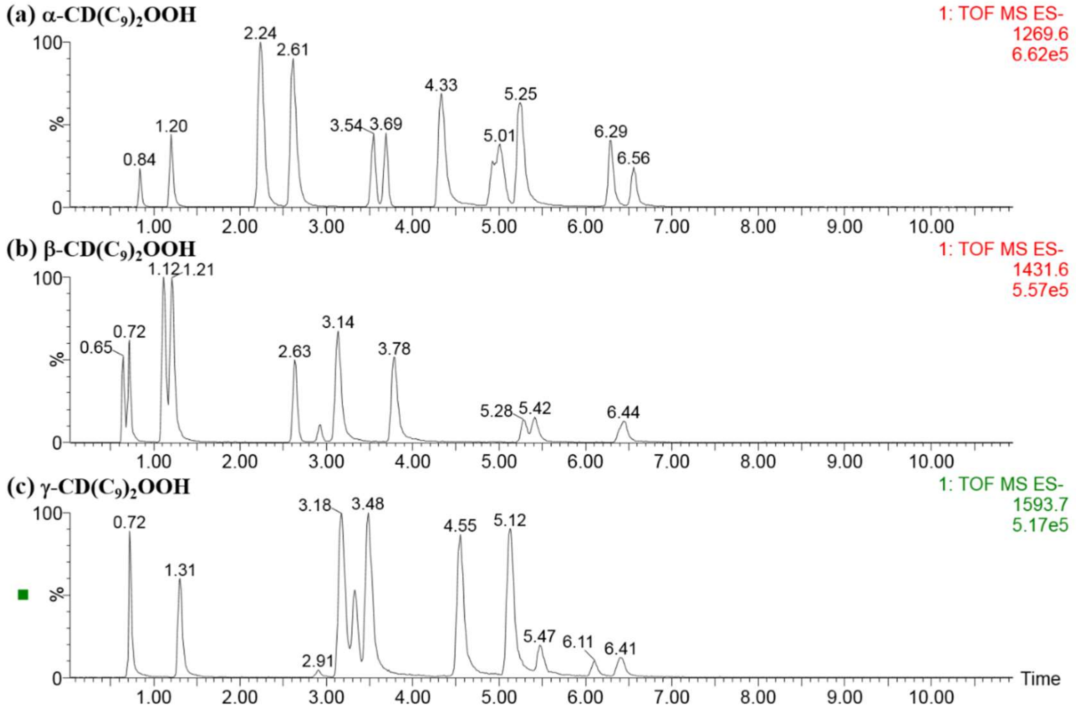

Figure S23 : [M-H]- reconstituted ion chromatograms of **7** : DS=1 m/z 1269.56 **(a)**, **8** DS=1 m/z 1431.61 **(b)** and **9** DS=1 m/z 1594.67 **(c)**.

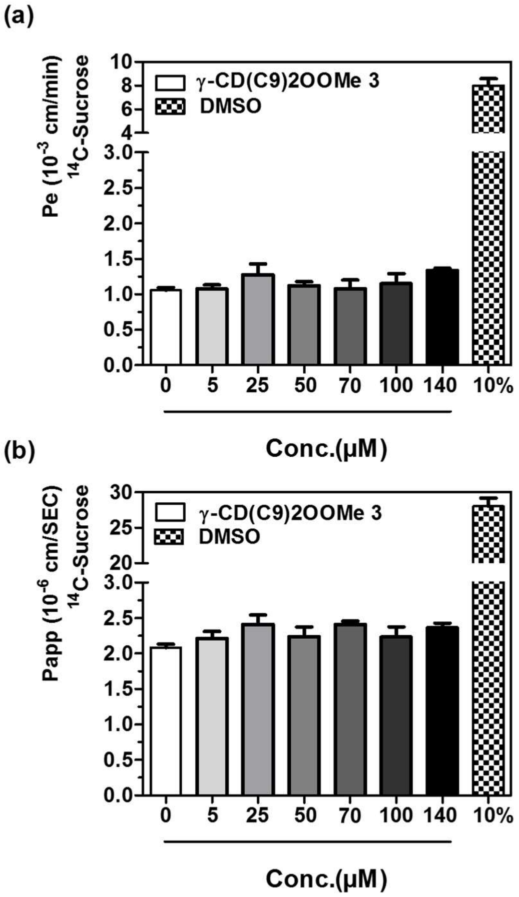

Figure S24: ^{14}C-Sucrose permeability (Pe) and apparent permeability (Papp) assessment in two in vitro models treated with different concentrations of **3**. DMSO 10% was used as positive control of biological barrier disruptions. Bars represent the mean of 6 filters + standard error of the mean. (a) represents the Pe of ^{14}C-Sucrose in the BLECs model and (b) represents the Papp of ^{14}C-Sucrose in the intestinal Caco-2 model

© 2020 by the authors. Submitted for possible open access publication under the terms and conditions of the Creative Commons Attribution (CC BY) license (http://creativecommons.org/licenses/by/4.0/).
